# PARP Inhibitor Olaparib and Its Combination Therapy in Metastatic Castration-resistant Prostate Cancer: A Systematic Review and Network Meta-analysis

**DOI:** 10.1016/j.euros.2025.12.014

**Published:** 2025-12-31

**Authors:** Yixian Li, Zongyu Li, Hongxia Lu, Pengjie Shi, Yiting Liu, Lilong Liu, Ke Chen

**Affiliations:** a Department of Urology, Tongji Hospital of Tongji Medical College, Huazhong University of Science and Technology, Wuhan, Hubei, China; b City University of Wuhan, Wuhan, Hubei, China

**Keywords:** Olaparib, Abiraterone, Metastatic castration-resistant prostate cancer, BRCA mutated, Homologous recombination repair

## Abstract

**Background and objective:**

Olaparib is one of the earliest approved treatment options for metastatic castration-resistant prostate cancer (mCRPC). This systematic review and network meta-analysis aimed to determine the optimal olaparib strategy for treating mCRPC.

**Methods:**

The Cochrane, Embase, PubMed, and Web of Science databases were searched comprehensively using “mCRPC” and “olaparib” as keywords. Study quality was appraised with the National Institutes of Health tools. Data were analyzed in R version 4.4.1. The primary endpoints included progression-free (PFS) and overall (OS) survival. The secondary endpoints included adverse events and severe adverse events (grade ≥3). Effect sizes were reported as hazard ratios (HRs) and risk ratios, with 95% credibility intervals (CrIs).

**Key findings and limitations:**

Nine studies from seven clinical trials involving 2355 patients were identified. For homologous recombination repair–mutated mCRPC, combination therapies did not demonstrate significant benefits compared with olaparib alone. However, for BRCA-mutated mCRPC, olaparib combined with abiraterone improved PFS (HR = 0.61, 95% CrI = 0.41–0.91) and OS (HR = 0.41, 95% CrI = 0.21–0.80) significantly. These significant advantages of olaparib combined with abiraterone were also observed in patients from different prostate-specific antigen subgroups.

**Conclusions and clinical implications:**

These findings suggest that olaparib combined with abiraterone offers substantial benefits in BRCA mutated type (BRCAmt) mCRPC patients. For those with BRCA wild type homologous recombination repair–mutated mCRPC, olaparib monotherapy is effective.

**Patient summary:**

We reviewed the published studies comparing different treatment options using the drug olaparib (alone or combined with other therapies) for advanced prostate cancer that has spread and no longer responds to standard hormone therapy (metastatic castration-resistant prostate cancer). We found evidence that the effectiveness of olaparib depends significantly on specific genetic features of the cancer. For patients whose cancer has changes in the *BRCA* genes, the combination of olaparib and the drug abiraterone was more effective in delaying cancer growth and improving survival than olaparib alone. For patients with changes in other related DNA repair genes (but not *BRCA*), olaparib alone was an effective treatment. This information may assist doctors and patients in choosing the most suitable treatment based on the cancer’s genetic characteristics.

## Take Home Message

For BRCA mutated–type metastatic castration-resistant prostate cancer (mCRPC), olaparib + abiraterone significantly improves progression-free or overall survival. In patients with HRR mutated-type/BRCA wild-type mCRPC, olaparib monotherapy suffices. Molecular subtyping is essential for optimal treatment selection.

## 1 Introduction

Prostate cancer is the second most frequently diagnosed malignancy and the sixth leading cause of cancer-related death in men worldwide [1]. Despite effective initial treatments, ∼10–20% of patients develop castration-resistant prostate cancer (CRPC) within 5 yr, and >80% of these individuals exhibit metastases at diagnosis [2]. The transition to metastatic CRPC (mCRPC) represents a fatal disease stage, as the 5-yr relative survival rate drops from nearly 100% for localized tumors to only 37% for those with distant metastases [3]. Approximately 25% of the affected individuals have mCRPC harboring mutations in homologous recombination repair (HRR) genes [4], [5], among which BRCA1/2 alterations are most frequent and correlate with notably worse outcomes [6], [7]. Although first-line treatment, such as abiraterone in the COU-AA-302 trial and enzalutamide in the PREVAIL trial, established median overall survival (OS) of ∼35 mo for chemotherapy-naïve mCRPC patients, real-world evidence often suggests that outcomes can be less favorable [8], [9], [10], [11], [12]. Consequently, it is imperative to develop novel therapeutic strategies, such as precision medicine approaches.

Olaparib is one of the earliest PARP inhibitors (PARPis) approved for the treatment of mCRPC. Multiple studies evaluating olaparib alone or in combination with other drugs, such as abiraterone, pembrolizumab, enzalutamide, or cediranib, have been completed and supported by preclinical evidence [13], [14], [15], [16], [17], [18]. However, the approved indications for olaparib-based therapies in mCRPC exhibit significant divergence between major regulatory bodies, particularly the U.S. Food and Drug Administration (FDA) and the European Medicines Agency (EMA), creating a complex landscape for clinical decision-making. For olaparib monotherapy (approved based on the PROfound trial), the FDA permits its use in patients with mCRPC harboring deleterious or suspected deleterious mutations in a broad panel of HRR genes, following progression on prior novel hormonal agents (NHAs) such as enzalutamide and abiraterone. In contrast, the EMA’s approval for monotherapy is more restrictive, limited to patients with BRCA1/2 mutations only. Furthermore, for the combination of olaparib and abiraterone (approved based on the PROpel trial), the FDA’s approval is narrowly indicated for patients with deleterious or suspected deleterious BRCA-mutated mCRPC. Conversely, the EMA has granted a broader approval for this combination in adult men with mCRPC for whom chemotherapy is not clinically indicated, irrespective of their biomarker status. These discrepancies underscore the ongoing debate about the patient populations that benefit most from these regimens and highlight the critical need for comparative evidence, which this network meta-analysis (NMA) aims to provide [19], [20], [21]. This difference indicates a lack of comparative data among olaparib-based regimens for optimal clinical decision-making.

An NMA integrates direct and indirect evidence to evaluate multiple interventions simultaneously. This study collected and stratified relevant data by HRR mutation status, BRCA mutation status, and baseline prostate-specific antigen (PSA) levels. This NMA was performed to compare the efficacy of olaparib monotherapy with that of various olaparib-based combination regimens, aiming to identify the patient populations most likely to benefit from these treatments.

## 2 Methods

This study followed Preferred Reporting Items for Systematic Reviews and Meta-analyses incorporating NMA guidelines [19] and was prospectively registered in the International Prospective Register of Systematic Reviews (CRD42024544254).

### 2.1 Literature search

A comprehensive search of Cochrane, Embase, PubMed, and Web of Science was performed using “mCRPC,” “olaparib,” and additional terms. The complete search strategy is listed in Supplementary Table 4. The search concluded on October 31, 2024. There were no language restrictions, although any article in a language other than English needed to include a requisite English abstract.

### 2.2 Study selection

Two reviewers independently evaluated all records, with a third reviewer synthesizing the screening results. Studies met the inclusion criteria if these: (1) included patients with mCRPC; (2) investigated olaparib monotherapy or combination therapy; (3) used next-generation hormone inhibitors (NHAs, such as abiraterone or enzalutamide), placebo, or other treatments as comparators; (4) reported primary endpoints, that is, progression-free survival (PFS) and OS, and secondary endpoints, that is, adverse events (AEs) and severe adverse events (SAEs, grade ≥3); and (5) employed controlled trial or cohort study design.

### 2.3 Risk of bias assessment

Two reviewers independently assessed the risk of bias, and a third reviewer synthesized the evaluations. The National Institutes of Health (NIH) quality assessment tools (QATs) were employed for this purpose [20]. Given that the NMA included studies with different designs—randomized controlled trials (RCTs) and retrospective cohort studies—two corresponding NIH QATs were used: the NIH Quality Assessment of Controlled Intervention Studies for RCTs and the NIH Quality Assessment Tool for Observational Cohort and Cross-sectional Studies for retrospective studies. The QAT results were used to identify the major sources of bias and guide the sensitivity analyses.

Specifically, sensitivity analyses were performed by excluding all nonprospective studies to test the robustness of the NMA’s conclusions against biases from lower-quality designs. The risk of bias was categorized as good, fair, or poor, and detailed results are presented in Supplementary Table 2.

### 2.4 Data extraction

Two reviewers independently extracted the data, with a third reviewer addressing any discrepancies. Using a standardized form, we collected study registration number, first author, year of publication, patient characteristics (age, PSA level, and Eastern Cooperative Oncology Group [ECOG] performance status), sample size, treatment regimens, and outcome measures.

### 2.5 Statistical analysis

All statistical analyses were conducted in R version 4.4.1 using the gemtc package (version 1.0-2; R Foundation for Statistical Computing, Vienna, Austria). The mtc.network function was applied to construct a network structure. The mtc.nodesplit and mtc.anohe functions were utilized to evaluate inconsistency (*p* < 0.05). Network structures that failed to meet the criteria for consistency or homogeneity were excluded from the Bayesian meta-analysis. To ensure methodological robustness, a complementary frequentist NMA was conducted using the netmeta package. The estimated heterogeneity variance (τ^2^) is reported in the Supplementary material, and the results of both statistical frameworks were compared for concordance. For time-to-event outcomes (PFS and OS), event probabilities at fixed time points were modeled using a random-effect model with a binomial likelihood and a complementary log-log (cloglog) link function. For dichotomous AE data (AEs/SAEs), relative risks (RRs) were estimated using a binomial likelihood and a log link function. All Bayesian analyses were executed in R version 4.4.1 with gemtc. A weakly informative prior from a normal distribution (mean = 0, precision = 0.0001; dnorm[0, 0.0001]) was used for relative treatment effects. This prior, corresponding to a standard deviation of approximately 100, minimizes prior influence while preventing implausible effect sizes. The heterogeneity parameter (τ) was modeled on its standard deviation scale with a uniform prior (dunif[0, om.scale]). Analyses were implemented using the mtc.run function, and convergence was assessed by the potential scale reduction factor, which was considered satisfactory when equal to 1.

Results were depicted through forest plots, league tables, and the surface under the cumulative ranking curve (SUCRA). Larger SUCRA values indicated higher ranking of the interventions.

To assess the robustness of our primary findings and explore the sources of heterogeneity, we conducted the following prespecified subgroup analyses. Subgroup analyses were performed for both the primary and the secondary outcomes, classified by HRR or BRCA mutation status (HRR wild type [HRRwt], HRR mutated type [HRRmt], BRCA wild type [BRCAwt], and BRCA mutated type [BRCAmt]). However, due to incomplete treatment regimens in the HRRwt and BRCAwt groups, only exploratory evaluations were performed, offering preliminary insights for subsequent research. Given substantial baseline PSA variations, we conducted subgroup analyses for the primary outcomes by stratifying patients into high (>100 μg/l) and low (≤100 μg/l) PSA groups. Baseline PSA data required for this subgroup analysis were consistently available and extracted from all trials included in our NMA. In the low PSA group (≤100 µg/l), due to inadequate data on treatment regimens, only an exploratory analysis was conducted. Additionally, to further mitigate the methodological bias inherent in open-label and retrospective study designs (eg, a selection or an information bias), a sensitivity analysis was performed by excluding all nonprospective studies.

As a complement to our primary Bayesian NMA, we conducted a complementary frequentist NMA. The purpose of this analysis was to ensure methodological robustness and confirm high consistency of results across different statistical frameworks.

## 3 Results

### 3.1 Literature screening

**Fig. 1:**
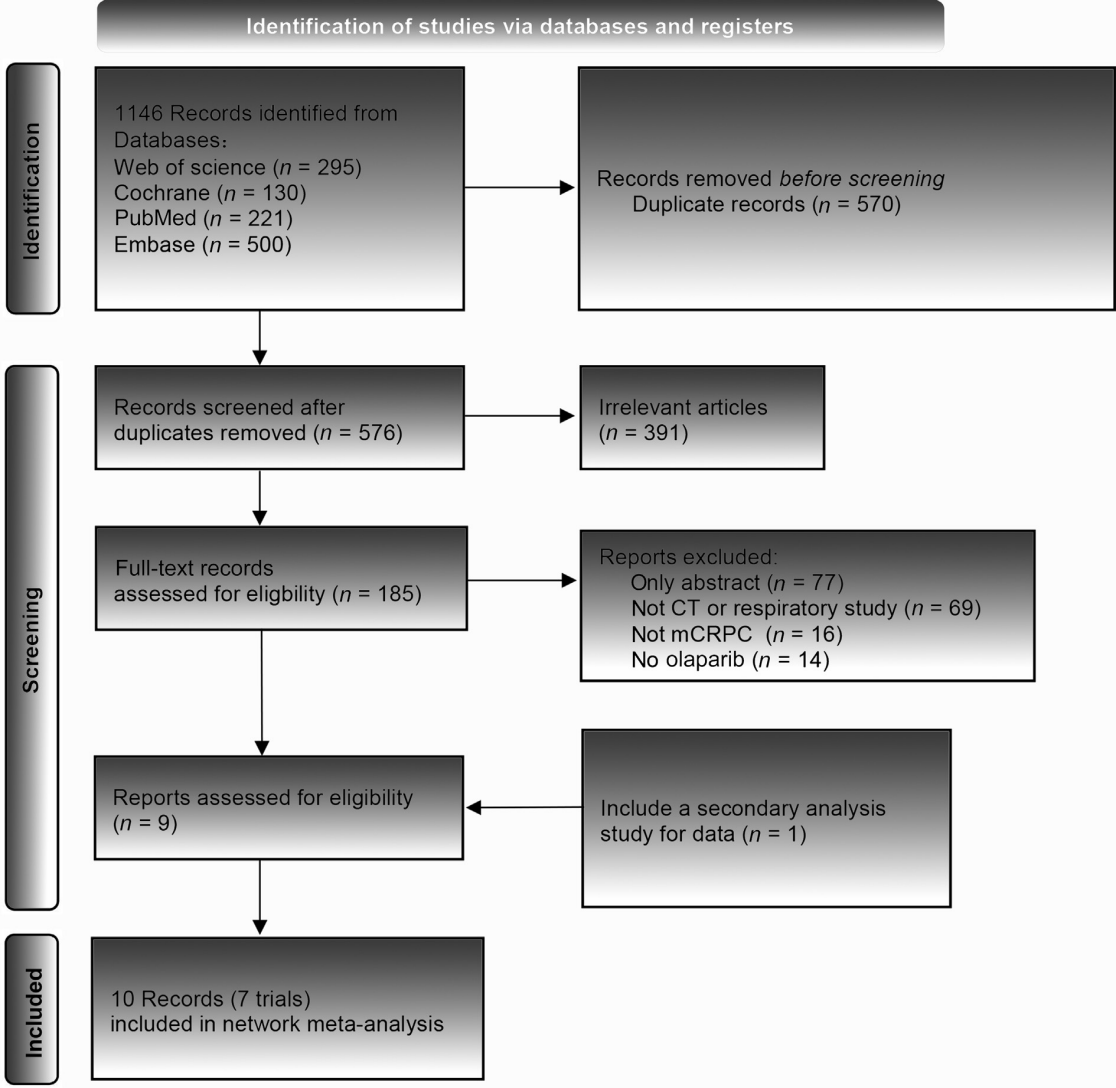
PRISMA flowchart of literature retrieving. CT = computed tomography; mCRPC = metastatic castration-resistant prostate cancer; PRISMA = Preferred Reporting Items for Systematic Reviews and Meta-analyses.

The initial search yielded 1146 records. After removing 570 duplicate entries, the titles and abstracts of 576 articles were screened, and 391 irrelevant articles were deleted. Full texts of the remaining 213 articles were checked to exclude 77 conference abstracts, 69 nonclinical controlled or retrospective studies, 16 non-mCRPC pathology studies, and 14 studies without olaparib treatment or single-arm design. The final analysis included ten articles describing seven clinical trials (Fig. 1).

### 3.2 Study characteristics

#### 3.2.1 Quality and general information of studies

This NMA included ten articles covering seven clinical trials published before October 30, 2023. Nine articles described findings from seven clinical trials, and one additional article provided subgroup data from the PROpel trial and Study 8 (NCT01972217) [21]. The overall quality of studies was high, although some open-label and retrospective designs carried a higher risk of bias (Supplementary Table 2) [13], [15], [16], [18], [22], [23]. The analysis comprised 2355 patients with mCRPC, with 374 patients receiving olaparib monotherapy, 882 receiving NHAs such as enzalutamide or abiraterone as monotherapy, 45 receiving olaparib combined with cediranib, 525 receiving olaparib combined with abiraterone, and 529 receiving olaparib combined with pembrolizumab.

The mean age was 66.6 ± 2.03 yr. Given considerable interstudy heterogeneity in PSA levels (18.6–632 μg/l), subgroup analyses were conducted by PSA levels. Most participants (99.1%) had ECOG performance status of 0 or 1, with only 21 patients scoring 2 (Table 1).

**Table 1:** Basic information of trials included

Trial	Registration	Trial	Patient information					Sample size	Publication included
		information	Previous management	Age, mean, SD	PSA (μg/l), mean, SD	ECOG (0/1/2)	Treatment		
Study 8	NCT01972217	Randomized, double-blind, phase 2 trial	Patients with mCRPC, formerly received docetaxel	70.0, 2.11	89.2, 36.0	34/36/1	Olaparib + abiraterone	71	Clarke (2018) [14]
				67.1, 2.53	55.9, 37.5	38/30/1	NHA	71	
PROpel	NCT03732820	Randomized, double-blind, phase 3 trial	Patients with mCRPC, formerly received ADT or first-generation antiandrogen agents	68.9, 8.12	31.0, 45.3	286/112/0	Olaparib + abiraterone	399	Clarke (2022) [37]
				69.9, 7.11	25.9, 35.0	272/124/0	NHA	397	Saad (2023) [18]
PROfound	NCT02987543	Randomized, open-label, phase 3 trial	Patients with mCRPC, disease progressed on NHA	69.0, 7.82	132, 202	131/112/13	Olaparib	256	de Bono (2020) [16]
				68.9, 7.33	160, 217	55/71/4	NHA	131	Mateo (2024) [25]
KEYLYNJNK-010	NCT03834519	Randomized, open-label, phase 3 trial	Patients with mCRPC, progressed on or after abiraterone or enzalutamide (but not both) and docetaxel	70.8, 8.06	139, 822	255/272/2	Pembrolizumab + olaparib	529	Antonarakis (2023) [17]
				68.9, 6.20	155, 709	139/125/0	NHA	264	
BRCAAway	NCT03012321	Randomized, open-label, phase 2 trial	Patients with mCRPC, formerly received at least one prior therapy for mCRPC/nmCRPC/mHSPC	68.3, 9.54	20.5, 26.0	16/5/0	Olaparib + abiraterone	21	Hussain (2024) [26]
				68.7, 4.77	18.6, 20.1	15/6/0	Olaparib	21	
				64.1, 7.21	53.2, 104	10/9/0	NHA	19	
NA	NCT02893917	Randomized, open-label, phase 2 trial	Patients with mCRPC, formerly received at least one prior therapy for mCRPC	65.7, 7.49	345, 714	–	Cediranib + olaparib	45	Kim (2023) [15]
				69.4, 7.04	177, 330	–	Olaparib	45	
NA	NA	Retrospective cohort study	Patients with mCRPC, progressed after abiraterone or first- or second-line treatment for CRPC	63.7, 8.85	632, 1193	18/16/0	Olaparib + abiraterone	34	Xie (2024) [13]
				67.0, 7.97	326, 625	26/26/0	Olaparib	52	

ADT = androgen deprivation therapy; CRPC = castration-resistant prostate cancer; ECOG = Eastern Cooperative Oncology Group; mCRPC = metastatic castration-resistant prostate cancer; mHSPC = metastatic hormone-sensitive prostate cancer; NA = not available; NHA = next-generation hormone inhibitor (abiraterone or enzalutamide); nmCRPC = nonmetastatic castration-resistant prostate cancer; PSA = prostate-specific antigen; SD = standard deviation.

#### 3.2.2 Progression-free survival

**Fig. 2:**
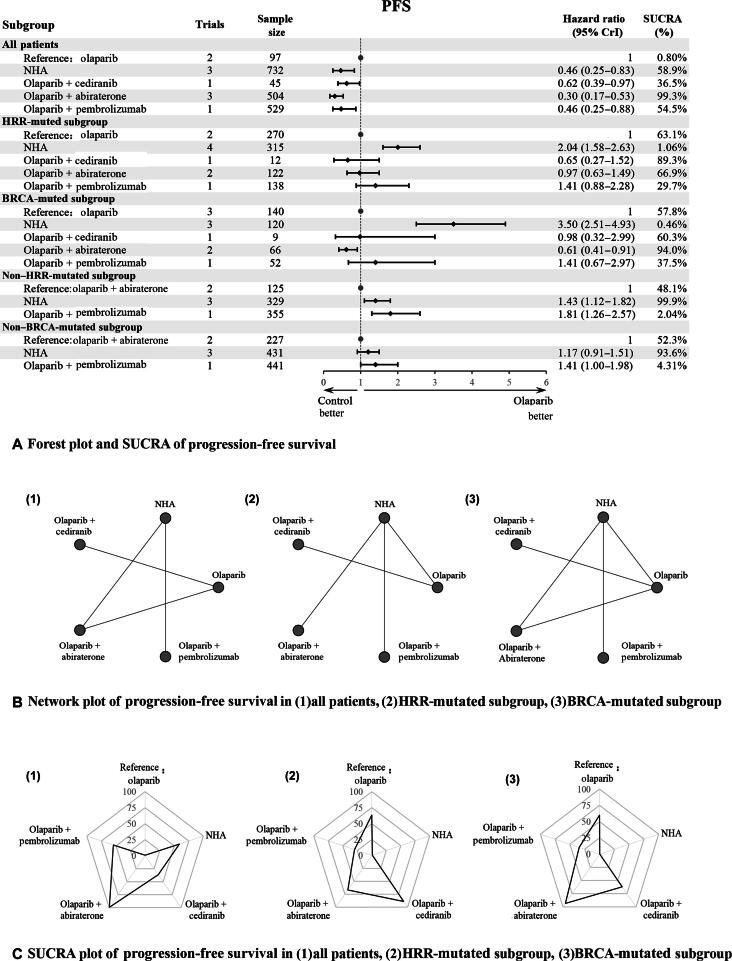
PFS plots in different gene-mutated subgroups: (A) forest plot and SUCRA of progression-free survival; (B) network plot of progression-free survival in (1) all patients, (2) HRR-mutated subgroup, and (3) BRCA-mutated subgroup; and (C) SUCRA plot of progression-free survival in (1) all patients, (2) HRR-mutated subgroup, and (3) BRCA-mutated subgroup. CrI = credibility interval; HRR = homologous recombination repair; NHA = novel hormonal agent; PFS = progression-free survival; SUCRA = surface under the cumulative ranking curve.

Seven studies, encompassing 2355 patients, provided data on PFS, which was one of the primary endpoints. In subgroup analyses stratified by genetic mutation status, five interventions were evaluated (Fig. 2B). Heterogeneity tests showed that the homogeneity assumption was satisfied across the NMA (Supplementary Fig. 1–5). Inconsistency tests using the node-splitting method were feasible only in the BRCAmt subgroup due to the absence of closed loops in other groups (Supplementary Fig. 11).

The analysis of the overall cohort, without distinguishing HRRmt from HRRwt, revealed that all four combination therapies offered statistically significant advantages over olaparib alone. Among these interventions, olaparib combined with abiraterone provided the most substantial benefit (hazard ratio [HR] = 0.30, 95% credibility interval [CrI] = 0.17–0.53). In the subgroup analysis by HRR mutation, NHAs (HR = 2.04, 95% CrI = 1.58–2.63) were significantly less effective than olaparib alone in the HRRmt subgroup, whereas the other combination regimens showed no significant differences from olaparib monotherapy. An exploratory analysis in the HRRwt subgroup, which comprised three interventions, indicated that olaparib combined with abiraterone was notably more effective than both NHAs (HR = 1.43, 95% CrI = 1.12–1.82) and olaparib combined with pembrolizumab (HR = 1.81, 95% CrI = 1.26–2.57).

A subgroup analysis based on BRCA status revealed that, in the BRCAmt group, NHAs (HR = 3.50, 95% CrI = 2.51–4.93) were significantly less effective than olaparib monotherapy. By contrast, olaparib combined with abiraterone (HR = 0.62, 95% CrI = 0.39–0.97) was more effective than olaparib alone, whereas other combination regimens showed no significant differences from olaparib. In the exploratory analysis of the BRCAwt group, olaparib combined with abiraterone showed a certain degree of advantage compared with both NHAs (HR = 1.17, 95% CrI = 0.91–1.51) and olaparib combined with pembrolizumab (HR = 1.41, 95% CrI = 1.00–1.98), although these differences were not statistically significant (Fig. 2A).

SUCRA rankings identified the optimal treatments: olaparib combined with abiraterone in the overall cohort (99.3%), olaparib combined with cediranib in the HRRmt group (89.2%), and olaparib combined with abiraterone in the BRCAmt group (94.0%; Fig. 2, Fig. 2). The findings from the league table (Supplementary Table 2) corroborated these SUCRA rankings.

In subgroup analyses based on PSA levels, five interventions were evaluated. In the subgroup with PSA >100 µg/l, both olaparib combined with cediranib (HR = 0.62, 95% CrI = 0.39–0.97) and olaparib combined with abiraterone (HR = 0.30, 95% CrI = 0.17–0.53) offered clear advantages over olaparib alone, whereas NHAs (HR = 2.00, 95% CrI = 1.60–2.60) and olaparib combined with pembrolizumab (HR = 2.10, 95% CrI = 1.50–2.90) were significantly less effective than olaparib monotherapy (Supplementary Fig. 13). In the subgroup with PSA <100 µg/l, only olaparib, NHAs, and olaparib combined with abiraterone were included in the exploratory analysis. Olaparib combined with abiraterone (HR = 0.37, 95% CrI = 0.17–0.82) provided a clear benefit over olaparib alone. No significant difference emerged between NHAs and olaparib. The findings from the league table (Supplementary Table 3) corroborated these SUCRA rankings.

#### 3.2.3 Overall survival

**Fig. 3:**
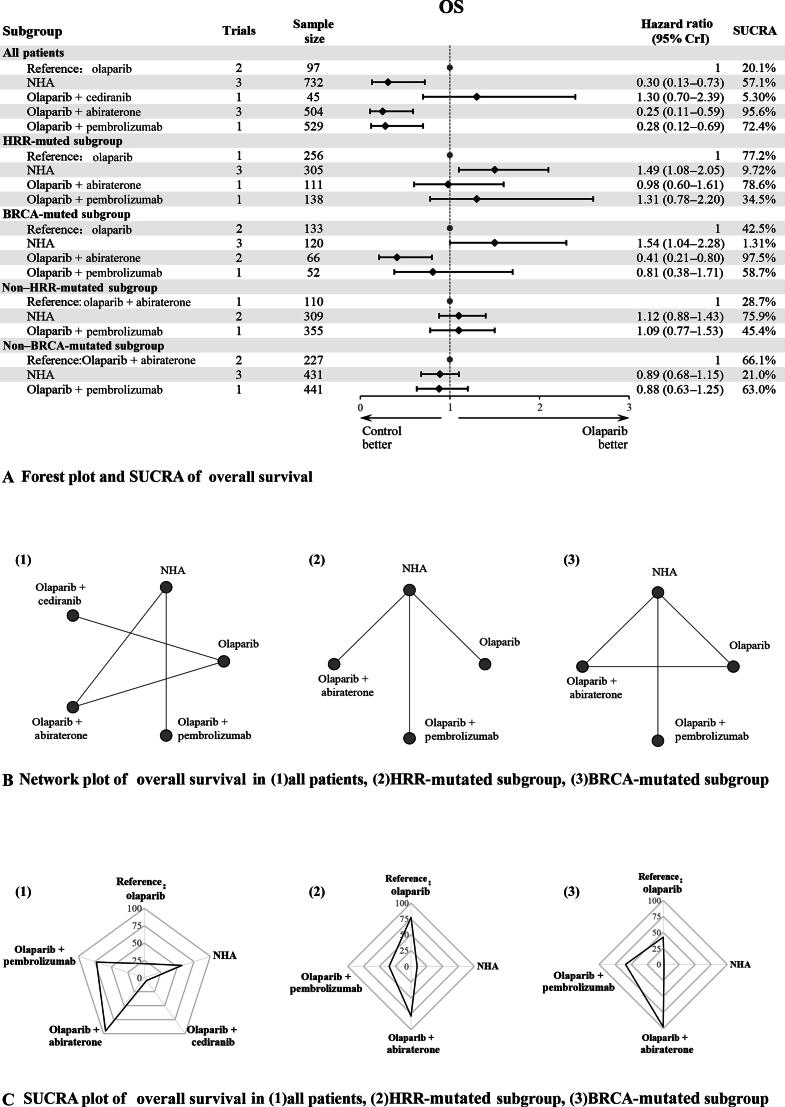
OS plots in different gene-mutated subgroups: (A) forest plot and SUCRA of overall survival; (B) network plot of overall survival in (1) all patients, (2) HRR-mutated subgroup, and (3) BRCA-mutated subgroup; and (C) SUCRA plot of overall survival in (1) all patients, (2) HRR-mutated subgroup, and (3) BRCA-mutated subgroup. CrI = credibility interval; HRR = homologous recombination repair; NHA = novel hormonal agent; OS = overall survival; SUCRA = surface under the cumulative ranking curve.

Six studies, including 2294 patients, provided data on OS, another primary endpoint. Five interventions were evaluated (Fig. 3B). Heterogeneity tests supported the homogeneity assumption across the NMA (Supplementary Fig. 6–8). Inconsistency tests using the node-splitting method were feasible only in the BRCAmt subgroup due to the absence of closed loops in other groups (Supplementary Fig. 12).

In the overall patient cohort, which was not stratified by genetic mutation status, three interventions, including NHAs (HR = 0.30, 95% CrI = 0.13–0.73), olaparib combined with abiraterone (HR = 0.25, 95% CrI = 0.11–0.59), and olaparib combined with pembrolizumab (HR = 0.28, 95% CrI 0.12–0.69), were significantly more effective than olaparib monotherapy. Olaparib combined with cediranib was slightly less effective than olaparib alone, although this difference did not reach statistical significance.

In the HRRmt subgroup, four interventions were evaluated. Compared with olaparib monotherapy, NHAs (HR = 1.49, 95% CrI = 1.08–2.05) were significantly less effective, while the other interventions did not differ significantly from olaparib. In the BRCAmt subgroup, NHAs (HR = 1.54, 95% CrI = 1.04–2.28) were also less effective than olaparib, whereas olaparib combined with abiraterone (HR = 0.41, 95% CrI = 0.21–0.80) was significantly more effective. Exploratory analyses in the HRRwt and BRCAwt subgroups revealed no statistically significant differences between the three interventions (Fig. 3A).

SUCRA rankings identified olaparib combined with abiraterone as the most effective intervention for the overall patient group (95.6%), and the HRRmt (77.2%) and BRCAmt (97.5%) subgroups (Fig. 3, Fig. 3). Supplementary Table 2 presents the league table for interventions and subgroup comparisons. SUCRA rankings were consistent with the results of the league table.

A subgroup analysis based on PSA levels included five interventions. In the subgroup with PSA >100 µg/l, olaparib combined with abiraterone (HR = 0.25, 95% CrI = 0.11–0.59) was significantly more effective than olaparib alone, whereas NHAs (HR = 1.50, 95% CrI = 1.10–2.10) were significantly less effective (Supplementary Fig. 13). In the subgroup with PSA <100 µg/l, only three interventions, including NHAs, olaparib, and olaparib combined with abiraterone, were included, and no exploratory analyses were conducted. Supplementary Table 3 present the league table of interventions and subgroup comparisons. SUCRA rankings were consistent with the league table results.

#### 3.2.4 Adverse events/severe adverse events

**Fig. 4:**
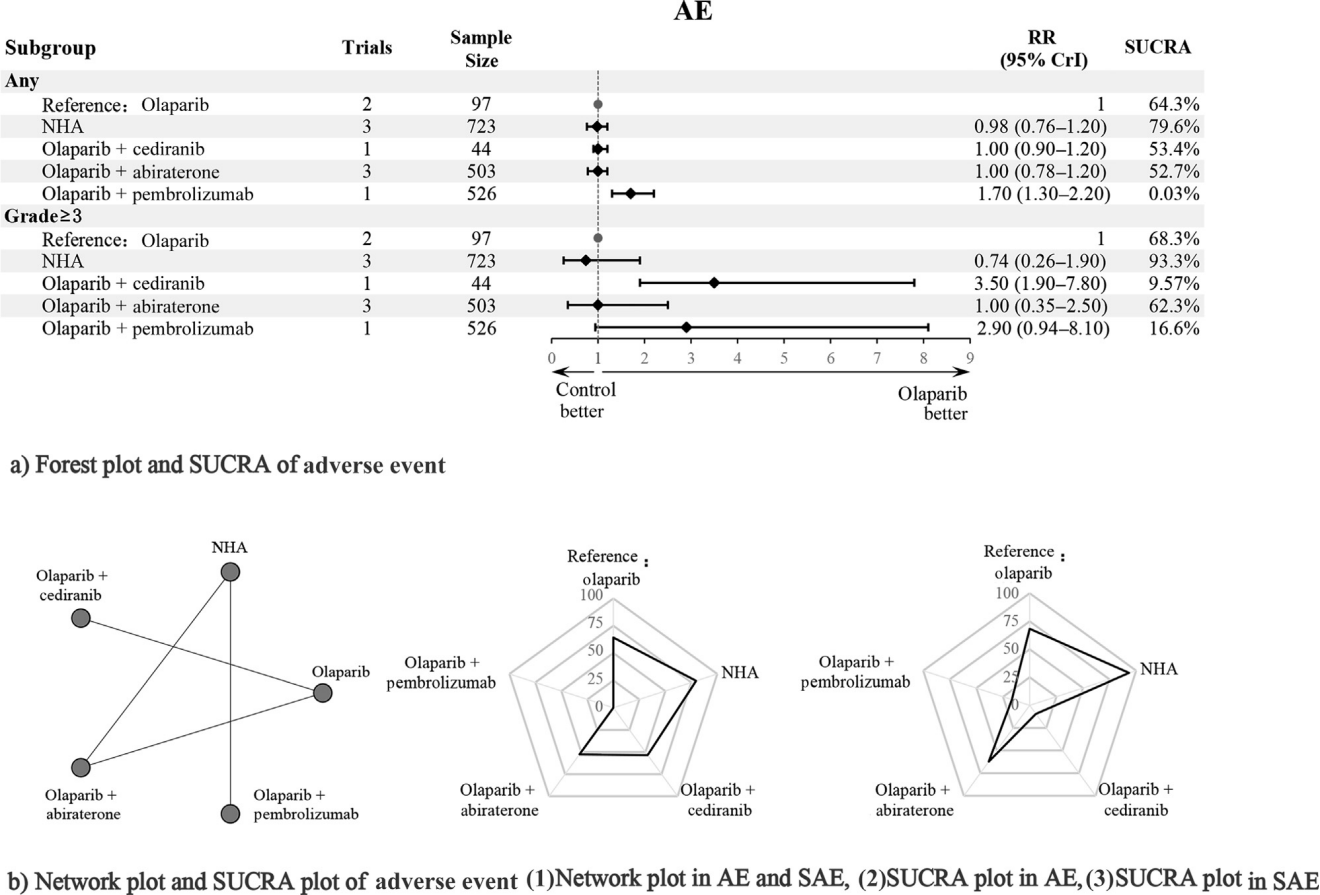
AE plots in different subgroups: (A) forest plot and SUCRA of adverse events, and (B) network and SUCRA plots of adverse event—(1) network plot for AEs and SAEs, (2) SUCRA plot for AEs, and (3) SUCRA plot for SAEs. AE = adverse event; CrI = credibility interval; NHA = novel hormonal agent; RR = relative risk; SAE = severe adverse event; SUCRA = surface under the cumulative ranking curve.

Six studies, including 2294 patients, reported the secondary endpoints—AEs and SAEs. Five interventions were evaluated (Fig. 4B). Heterogeneity tests supported the homogeneity assumption across the NMA (Supplementary Fig. 9–10). Inconsistency tests using the node-splitting method were not feasible due to no closed loops in the network structure.

In the overall population, which was not stratified by genetic mutation status, no significant differences in the risk of AEs were found when NHAs, olaparib combined with cediranib, or olaparib combined with abiraterone was compared with olaparib monotherapy. However, olaparib combined with pembrolizumab (RR = 1.70, 95% CrI = 1.30–2.20) was notably less favorable than olaparib alone. For SAEs, NHAs and olaparib combined with abiraterone did not differ significantly from olaparib monotherapy, whereas olaparib combined with pembrolizumab (RR = 2.90, 95% CrI = 0.94–8.10) was moderately less favorable, and olaparib combined with cediranib (RR = 3.50, 95% CrI = 1.90–7.80) was substantially less favorable (Fig. 4A).

SUCRA values identified NHAs as the most favorable intervention for both AEs (79.6%) and SAEs (93.3%; Fig. 4, Fig. 4). The league table for interventions and subgroup comparisons is shown (Supplementary Table 2). SUCRA rankings corroborated the league table results.

## 4 Discussion

This study included several pioneering investigations of olaparib and its combination therapies, and the results align with the findings from preclinical and prior clinical research [9]. In the overall patient population, cediranib was effective in improving radiographic PFS, while its impact on OS was limited compared with olaparib. The higher incidence of grade ≥3 AEs with cediranib [15] indicates its limited utility in the broader mCRPC population. Olaparib combined with pembrolizumab improved both PFS and OS significantly but increased toxicities [18], necessitating careful patient selection based on individual clinical characteristics. In contrast, olaparib combined with abiraterone demonstrated superior PFS and OS outcomes to olaparib monotherapy without increasing the risk of AEs. These findings suggest that olaparib combined with abiraterone is an effective strategy for mCRPC treatment. However, it is important to note that treatment efficacy varies significantly with individual genetic factors, making molecular testing essential for optimal treatment selection.

In subgroup analyses by mutation status, NHAs were significantly less effective than olaparib and other regimens in both HRRmt and BRCAmt populations. Olaparib alone remained sufficiently efficacious in the HRRmt subgroup, and combination therapies did not confer significant benefits. In the BRCAmt subgroup, olaparib combined with abiraterone significantly improved both PFS and OS compared with olaparib alone. Exploratory analyses in HRRwt and BRCAwt populations demonstrated distinct outcomes. In HRRwt patients, olaparib combined with abiraterone significantly improved PFS versus NHAs and olaparib combined with pembrolizumab, with no significant OS differences. In the BRCAwt population, neither PFS nor OS showed distinct advantages across treatments. A subgroup analysis by PSA levels revealed that olaparib combined with abiraterone was superior to olaparib alone in all PSA subgroups. These findings imply that NHAs are not the most suitable choice for HRRmt or BRCAmt patients. Olaparib appears most appropriate for HRRmt-BRCAwt cases, while olaparib combined with abiraterone is optimal for BRCAmt patients. Further studies should evaluate the efficacy of these treatments in HRRwt or BRCAwt patients.

Preclinical evidence indicates that the effectiveness of combining PARPis with androgen receptor (AR) pathway inhibitors (ARPIs) relies on synthetic lethality, independent of the synergistic patterns involving HRR mutations. Androgen deprivation therapy heightens tumor sensitivity to PARP treatment [24], [25], [26], and PARP enzymatic activity is associated with AR function. Moreover, a PARPi suppresses not only PARP itself, but also AR activity [27], [28]. The robust PARP-trapping activity of olaparib may account for the superior performance of olaparib combined with abiraterone. PARPi agents with less potent trapping capabilities have shown markedly inferior outcomes in combination regimens [9], [24], [29], [30], [31], [32]. Furthermore, olaparib combined with abiraterone does not exacerbate drug-related toxicities or compromise the quality of life [11], [32], [33], [34], [35], [36]. Nevertheless, FDA and EMA criteria differ in determining the most suitable patient populations for olaparib monotherapy, olaparib combined with abiraterone, and other PARP combined with ARPI combinations [37], [38], [39]. The current results align with the secondary analyses from Study 8 (NCT01972217) and PROpel trials [21], supporting FDA approval of olaparib combined with abiraterone for BRCAmt patients. However, the comparative efficacy of olaparib and combination therapies in HRRwt patients remains unclear. The TOPARP-A trial and other clinical studies [29], [40], [41] have shown that olaparib benefits in mCRPC were limited to HRRmt patients; yet, this NMA and two other trials of PARPis combined with NHAs (not included here) [42], [43] suggest potential benefits for HRRwt patients. Moreover, olaparib combined with NHAs showed fewer AEs, which is consistent with a previous report [9] but different from the TOPARP-A findings. Further clinical studies are needed to determine optimal first-line therapy for HRRwt patients.

Existing meta-analyses have focused primarily on direct comparisons of PARPi monotherapy with combination therapies [44], [45], including PARPis combined with ARPIs. Some studies have also compared PARPis with first-line regimens. For example, the efficacy of olaparib is comparable with that of platinum-based chemotherapy in BRCAmt patients [46]. To our knowledge, the present study is the first to comprehensively evaluate all olaparib-based treatments for mCRPC. Our subgroup analyses revealed potential differences in therapeutic responses across various patient subgroups. However, the number of eligible studies was limited, and not all interventions were represented across subgroups. Some subgroups included relatively small sample sizes, and an assessment of the publication bias was not performed. Standard approaches for publication bias detection, such as funnel plots, have limited statistical power and are unreliable when fewer than ten trials are available, as in the present network. Consequently, the overall findings require further validation. In addition, inconsistency testing was not feasible for several outcomes owing to the absence of closed loops within the evidence network.

Given the limited number of included trials, the current statistical analysis findings are preliminary, and further research is still desired to corroborate our conclusions. Ongoing clinical studies that group patients by genetic mutation subtypes, particularly with subgroup analyses of combination therapies by AEs, remain scarce. Current safety data on olaparib and its combination therapies are primarily limited to the overall study population, and more precise subgroup analyses could offer tailored guidance for specific genetic profiles. Larger, prestratified trials based on mutation status are needed. Furthermore, open-label designs may introduce performance and detection biases, as both participants and clinicians are aware of treatment allocation. Retrospective designs are also vulnerable to selection and information biases due to their nonprospective nature. Hence, the results of this analysis should be interpreted with caution. Future prospective, randomized, double-blind trials are required to confirm these findings.

## 5 Conclusions

This study suggests that olaparib monotherapy is most suitable for HRRmt-BRCAwt patients, whereas olaparib combined with abiraterone is the best option for those with BRCAmt. Given the limitations of this study, conducting additional studies is necessary, particularly clinical trials that prestratify participants by genetic mutation status, to identify optimal strategies for HRRwt patients.

  ***Author contributions*:** Lilong Liu had full access to all the data in the study and takes responsibility for the integrity of the data and the accuracy of the data analysis.

  *Study concept and design*: Y. Li, L. Liu.

*Acquisition of data*: Y. Liu, L. Liu.

*Analysis and interpretation of data*: Y. Li, Lu.

*Drafting of the manuscript*: Y. Li, L. Liu.

*Critical revision of the manuscript for important intellectual content*: Y. Li, Z. Li, Lu, Shi, Y. Liu.

*Statistical analysis*: Y. Li, Shi.

*Obtaining funding*: None.

*Administrative, technical, or material support*: Chen.

*Supervision*: Chen, L. Liu.

*Other*: None.

  ***Financial disclosures:*** Lilong Liu certifies that all conflicts of interest, including specific financial interests and relationships and affiliations relevant to the subject matter or materials discussed in the manuscript (eg, employment/affiliation, grants or funding, consultancies, honoraria, stock ownership or options, expert testimony, royalties, or patents filed, received, or pending), are the following: None.

  ***Funding/Support and role of the sponsor*:** None.

  ***Data sharing statement*:** The datasets used and analyzed during the current study are available from the corresponding author on reasonable request.
