# Supplementary material for: PARP Inhibitor Olaparib and Its Combination Therapy in Metastatic Castration-resistant Prostate Cancer: A Systematic Review and Network Meta-analysis
Source: Eur Urol Open Sci. 2025 Dec 31;84:1–12. doi: 10.1016/j.euros.2025.12.014 (PMC12804617; doi:10.1016/j.euros.2025.12.014)

**Supplementary Table 1:** Search strategy.

| Pubmed | | |
| --- | --- | --- |
| # | Query | Results |
| 1 | (('androgen independent metastatic PC*') OR ('androgen independent metastatic prostate cancer') OR ('androgen independent metastatic prostatic cancer') OR ('Androgen Independent Prostatic Cancer*') OR ('Androgen Independent Prostatic Neoplasm*') OR ('androgen insensitive metastatic PC*') OR ('androgen insensitive metastatic prostate cancer') OR ('androgen insensitive metastatic prostatic cancer') OR ('Androgen Insensitive Prostatic Cancer*') OR ('Androgen Insensitive Prostatic Neoplasm*') OR ('Androgen Resistant Prostatic Cancer*') OR ('Androgen Resistant Prostatic Neoplasm*') OR ('castrate refractory metastatic PC*') OR ('castrate refractory metastatic prostate cancer') OR ('castrate refractory metastatic prostatic cancer') OR ('castrate resistant metastatic PC*') OR ('castrate resistant metastatic prostate cancer') OR ('castrate resistant metastatic prostatic cancer') OR ('castration refractory metastatic PC*') OR ('castration refractory metastatic prostate cancer') OR ('castration refractory metastatic prostatic cancer') OR ('castration resistant metastatic PC*') OR ('castration resistant metastatic prostate cancer') OR ('castration resistant metastatic prostatic cancer') OR ('castration resistant metastatic prostatic neoplasm') OR ('Castration Resistant Prostatic Cancer*') OR ('Castration Resistant Prostatic Neoplasm*') OR ('hormone refractory metastatic PC*') OR ('hormone refractory metastatic prostate cancer') OR ('hormone refractory metastatic prostatic cancer') OR ('Hormone Refractory Prostatic Cancer') OR ('Hormone Refractory Prostatic Neoplasms') OR ('hormone resistant metastatic PC*') OR ('hormone resistant metastatic prostate cancer') OR ('hormone resistant metastatic prostatic cancer') OR ('metastatic androgen independent PC*') OR ('metastatic androgen independent prostate cancer') OR ('metastatic androgen independent prostatic cancer') OR ('metastatic androgen insensitive prostate cancer') OR ('metastatic androgen insensitive prostatic cancer') OR ('metastatic castrate refractory PC*') OR ('metastatic castrate refractory prostate cancer') OR ('metastatic castrate refractory prostatic cancer') OR ('metastatic castrate resistant PC*') OR ('metastatic castrate resistant prostate cancer') OR ('metastatic castrate resistant prostatic cancer') OR ('metastatic castration refractory PC*') OR ('metastatic castration refractory prostate cancer') OR ('metastatic castration refractory prostatic cancer') OR ('metastatic castration resistant PC*') OR ('metastatic castration resistant prostate cancer') OR ('metastatic castration resistant prostatic cancer') OR ('metastatic castration resistant prostatic neoplasm') OR ('metastatic hormone refractory PC*') OR ('metastatic hormone refractory prostate cancer') OR ('metastatic hormone refractory prostatic cancer') OR ('metastatic hormone resistant PC*') OR ('metastatic hormone resistant prostate cancer') OR ('metastatic hormone resistant prostatic cancer') | 221 |
| 2 | ('azd 2281') OR ('AZD221') OR ('azd2281') OR ('AZD-2281') OR ('ku 0059436') OR ('ku 59436') OR ('ku0059436') OR ('ku59436') OR ('lynparza') OR ('mk 7339') OR ('mk7339') OR ('ng 1002') OR ('ng1002') OR ('olaparib') OR ('ro 8508245') OR ('ro8508245') ) |  |

| Embase | | |
| --- | --- | --- |
| # | Query | Results |
| 1 | ‘metastatic castration resistant prostate cancer’/exp OR ('androgen independent metastatic PC*' OR 'androgen independent metastatic prostate cancer' OR 'androgen independent metastatic prostatic cancer' OR 'Androgen Independent Prostatic Cancer*' OR 'Androgen Independent Prostatic Neoplasm*' OR 'androgen insensitive metastatic PC*' OR 'androgen insensitive metastatic prostate cancer' OR 'androgen insensitive metastatic prostatic cancer' OR 'Androgen Insensitive Prostatic Cancer*' OR 'Androgen Insensitive Prostatic Neoplasm*' OR 'Androgen Resistant Prostatic Cancer*' OR 'Androgen Resistant Prostatic Neoplasm*' OR 'castrate refractory metastatic PC*' OR 'castrate refractory metastatic prostate cancer' OR 'castrate refractory metastatic prostatic cancer' OR 'castrate resistant metastatic PC*' OR 'castrate resistant metastatic prostate cancer' OR 'castrate resistant metastatic prostatic cancer' OR 'castration refractory metastatic PC*' OR 'castration refractory metastatic prostate cancer' OR 'castration refractory metastatic prostatic cancer' OR 'castration resistant metastatic PC*' OR 'castration resistant metastatic prostate cancer' OR 'castration resistant metastatic prostatic cancer' OR 'castration resistant metastatic prostatic neoplasm' OR 'Castration Resistant Prostatic Cancer*' OR 'Castration Resistant Prostatic Neoplasm*' OR 'hormone refractory metastatic PC*' OR 'hormone refractory metastatic prostate cancer' OR 'hormone refractory metastatic prostatic cancer' OR 'Hormone Refractory Prostatic Cancer' OR 'Hormone Refractory Prostatic Neoplasms' OR 'hormone resistant metastatic PC*' OR 'hormone resistant metastatic prostate cancer' OR 'hormone resistant metastatic prostatic cancer' OR 'metastatic androgen independent PC*' OR 'metastatic androgen independent prostate cancer' OR 'metastatic androgen independent prostatic cancer' OR 'metastatic androgen insensitive prostate cancer' OR 'metastatic androgen insensitive prostatic cancer' OR 'metastatic castrate refractory PC*' OR 'metastatic castrate refractory prostate cancer' OR 'metastatic castrate refractory prostatic cancer' OR 'metastatic castrate resistant PC*' OR 'metastatic castrate resistant prostate cancer' OR 'metastatic castrate resistant prostatic cancer' OR 'metastatic castration refractory PC*' OR 'metastatic castration refractory prostate cancer' OR 'metastatic castration refractory prostatic cancer' OR 'metastatic castration resistant PC*' OR 'metastatic castration resistant prostate cancer' OR 'metastatic castration resistant prostatic cancer' OR 'metastatic castration resistant prostatic neoplasm' OR 'metastatic hormone refractory PC*' OR 'metastatic hormone refractory prostate cancer' OR 'metastatic hormone refractory prostatic cancer' OR 'metastatic hormone resistant PC*' OR 'metastatic hormone resistant prostate cancer' OR 'metastatic hormone resistant prostatic cancer'):ti,ab,kw | 500 |
| 2 | ‘Olaparib’/exp OR ('azd 2281' OR 'AZD221' OR 'azd2281' OR 'AZD-2281' OR 'ku 0059436' OR 'ku 59436' OR 'ku0059436' OR 'ku59436' OR 'lynparza' OR 'mk 7339' OR 'mk7339' OR 'ng 1002' OR 'ng1002' OR 'olaparib' OR 'ro 8508245' OR 'ro8508245'):ti,ab,kw |  |

| Cochrane Library | | |
| --- | --- | --- |
| # | Query | Results |
| 1 | (('androgen independent metastatic PC*') OR ('androgen independent metastatic prostate cancer') OR ('androgen independent metastatic prostatic cancer') OR ('Androgen Independent Prostatic Cancer*') OR ('Androgen Independent Prostatic Neoplasm*') OR ('androgen insensitive metastatic PC*') OR ('androgen insensitive metastatic prostate cancer') OR ('androgen insensitive metastatic prostatic cancer') OR ('Androgen Insensitive Prostatic Cancer*') OR ('Androgen Insensitive Prostatic Neoplasm*') OR ('Androgen Resistant Prostatic Cancer*') OR ('Androgen Resistant Prostatic Neoplasm*') OR ('castrate refractory metastatic PC*') OR ('castrate refractory metastatic prostate cancer') OR ('castrate refractory metastatic prostatic cancer') OR ('castrate resistant metastatic PC*') OR ('castrate resistant metastatic prostate cancer') OR ('castrate resistant metastatic prostatic cancer') OR ('castration refractory metastatic PC*') OR ('castration refractory metastatic prostate cancer') OR ('castration refractory metastatic prostatic cancer') OR ('castration resistant metastatic PC*') OR ('castration resistant metastatic prostate cancer') OR ('castration resistant metastatic prostatic cancer') OR ('castration resistant metastatic prostatic neoplasm') OR ('Castration Resistant Prostatic Cancer*') OR ('Castration Resistant Prostatic Neoplasm*') OR ('hormone refractory metastatic PC*') OR ('hormone refractory metastatic prostate cancer') OR ('hormone refractory metastatic prostatic cancer') OR ('Hormone Refractory Prostatic Cancer') OR ('Hormone Refractory Prostatic Neoplasms') OR ('hormone resistant metastatic PC*') OR ('hormone resistant metastatic prostate cancer') OR ('hormone resistant metastatic prostatic cancer') OR ('metastatic androgen independent PC*') OR ('metastatic androgen independent prostate cancer') OR ('metastatic androgen independent prostatic cancer') OR ('metastatic androgen insensitive prostate cancer') OR ('metastatic androgen insensitive prostatic cancer') OR ('metastatic castrate refractory PC*') OR ('metastatic castrate refractory prostate cancer') OR ('metastatic castrate refractory prostatic cancer') OR ('metastatic castrate resistant PC*') OR ('metastatic castrate resistant prostate cancer') OR ('metastatic castrate resistant prostatic cancer') OR ('metastatic castration refractory PC*') OR ('metastatic castration refractory prostate cancer') OR ('metastatic castration refractory prostatic cancer') OR ('metastatic castration resistant PC*') OR ('metastatic castration resistant prostate cancer') OR ('metastatic castration resistant prostatic cancer') OR ('metastatic castration resistant prostatic neoplasm') OR ('metastatic hormone refractory PC*') OR ('metastatic hormone refractory prostate cancer') OR ('metastatic hormone refractory prostatic cancer') OR ('metastatic hormone resistant PC*') OR ('metastatic hormone resistant prostate cancer') OR ('metastatic hormone resistant prostatic cancer') | 130 |
| 2 | ('azd 2281') OR ('AZD221') OR ('azd2281') OR ('AZD-2281') OR ('ku 0059436') OR ('ku 59436') OR ('ku0059436') OR ('ku59436') OR ('lynparza') OR ('mk 7339') OR ('mk7339') OR ('ng 1002') OR ('ng1002') OR ('olaparib') OR ('ro 8508245') OR ('ro8508245') ) |  |

| Web of Science | |
| --- | --- |
| Query | Results |
| (TI=(("androgen independent metastatic PC*") OR ("androgen independent metastatic prostate cancer") OR ("androgen independent metastatic prostatic cancer") OR ("Androgen Independent Prostatic Cancer*") OR ("Androgen Independent Prostatic Neoplasm*") OR ("androgen insensitive metastatic PC*") OR ("androgen insensitive metastatic prostate cancer") OR ("androgen insensitive metastatic prostatic cancer") OR ("Androgen Insensitive Prostatic Cancer*") OR ("Androgen Insensitive Prostatic Neoplasm*") OR ("Androgen Resistant Prostatic Cancer*") OR ("Androgen Resistant Prostatic Neoplasm*") OR ("castrate refractory metastatic PC*") OR ("castrate refractory metastatic prostate cancer") OR ("castrate refractory metastatic prostatic cancer") OR ("castrate resistant metastatic PC*") OR ("castrate resistant metastatic prostate cancer") OR ("castrate resistant metastatic prostatic cancer") OR ("castration refractory metastatic PC*") OR ("castration refractory metastatic prostate cancer") OR ("castration refractory metastatic prostatic cancer") OR ("castration resistant metastatic PC*") OR ("castration resistant metastatic prostate cancer") OR ("castration resistant metastatic prostatic cancer") OR ("castration resistant metastatic prostatic neoplasm") OR ("Castration Resistant Prostatic Cancer*") OR ("Castration Resistant Prostatic Neoplasm*") OR ("hormone refractory metastatic PC*") OR ("hormone refractory metastatic prostate cancer") OR ("hormone refractory metastatic prostatic cancer") OR ("Hormone Refractory Prostatic Cancer") OR ("Hormone Refractory Prostatic Neoplasms") OR ("hormone resistant metastatic PC*") OR ("hormone resistant metastatic prostate cancer") OR ("hormone resistant metastatic prostatic cancer") OR ("metastatic androgen independent PC*") OR ("metastatic androgen independent prostate cancer") OR ("metastatic androgen independent prostatic cancer") OR ("metastatic androgen insensitive prostate cancer") OR ("metastatic androgen insensitive prostatic cancer") OR ("metastatic castrate refractory PC*") OR ("metastatic castrate refractory prostate cancer") OR ("metastatic castrate refractory prostatic cancer") OR ("metastatic castrate resistant PC*") OR ("metastatic castrate resistant prostate cancer") OR ("metastatic castrate resistant prostatic cancer") OR ("metastatic castration refractory PC*") OR ("metastatic castration refractory prostate cancer") OR ("metastatic castration refractory prostatic cancer") OR ("metastatic castration resistant PC*") OR ("metastatic castration resistant prostate cancer") OR ("metastatic castration resistant prostatic cancer") OR ("metastatic castration resistant prostatic neoplasm") OR ("metastatic hormone refractory PC*") OR ("metastatic hormone refractory prostate cancer") OR ("metastatic hormone refractory prostatic cancer") OR ("metastatic hormone resistant PC*") OR ("metastatic hormone resistant prostate cancer") OR ("metastatic hormone resistant prostatic cancer")) OR AB=(("androgen independent metastatic PC*") OR ("androgen independent metastatic prostate cancer") OR ("androgen independent metastatic prostatic cancer") OR ("Androgen Independent Prostatic Cancer*") OR ("Androgen Independent Prostatic Neoplasm*") OR ("androgen insensitive metastatic PC*") OR ("androgen insensitive metastatic prostate cancer") OR ("androgen insensitive metastatic prostatic cancer") OR ("Androgen Insensitive Prostatic Cancer*") OR ("Androgen Insensitive Prostatic Neoplasm*") OR ("Androgen Resistant Prostatic Cancer*") OR ("Androgen Resistant Prostatic Neoplasm*") OR ("castrate refractory metastatic PC*") OR ("castrate refractory metastatic prostate cancer") OR ("castrate refractory metastatic prostatic cancer") OR ("castrate resistant metastatic PC*") OR ("castrate resistant metastatic prostate cancer") OR ("castrate resistant metastatic prostatic cancer") OR ("castration refractory metastatic PC*") OR ("castration refractory metastatic prostate cancer") OR ("castration refractory metastatic prostatic cancer") OR ("castration resistant metastatic PC*") OR ("castration resistant metastatic prostate cancer") OR ("castration resistant metastatic prostatic cancer") OR ("castration resistant metastatic prostatic neoplasm") OR ("Castration Resistant Prostatic Cancer*") OR ("Castration Resistant Prostatic Neoplasm*") OR ("hormone refractory metastatic PC*") OR ("hormone refractory metastatic prostate cancer") OR ("hormone refractory metastatic prostatic cancer") OR ("Hormone Refractory Prostatic Cancer") OR ("Hormone Refractory Prostatic Neoplasms") OR ("hormone resistant metastatic PC*") OR ("hormone resistant metastatic prostate cancer") OR ("hormone resistant metastatic prostatic cancer") OR ("metastatic androgen independent PC*") OR ("metastatic androgen independent prostate cancer") OR ("metastatic androgen independent prostatic cancer") OR ("metastatic androgen insensitive prostate cancer") OR ("metastatic androgen insensitive prostatic cancer") OR ("metastatic castrate refractory PC*") OR ("metastatic castrate refractory prostate cancer") OR ("metastatic castrate refractory prostatic cancer") OR ("metastatic castrate resistant PC*") OR ("metastatic castrate resistant prostate cancer") OR ("metastatic castrate resistant prostatic cancer") OR ("metastatic castration refractory PC*") OR ("metastatic castration refractory prostate cancer") OR ("metastatic castration refractory prostatic cancer") OR ("metastatic castration resistant PC*") OR ("metastatic castration resistant prostate cancer") OR ("metastatic castration resistant prostatic cancer") OR ("metastatic castration resistant prostatic neoplasm") OR ("metastatic hormone refractory PC*") OR ("metastatic hormone refractory prostate cancer") OR ("metastatic hormone refractory prostatic cancer") OR ("metastatic hormone resistant PC*") OR ("metastatic hormone resistant prostate cancer") OR ("metastatic hormone resistant prostatic cancer")) OR AK=(("androgen independent metastatic PC*") OR ("androgen independent metastatic prostate cancer") OR ("androgen independent metastatic prostatic cancer") OR ("Androgen Independent Prostatic Cancer*") OR ("Androgen Independent Prostatic Neoplasm*") OR ("androgen insensitive metastatic PC*") OR ("androgen insensitive metastatic prostate cancer") OR ("androgen insensitive metastatic prostatic cancer") OR ("Androgen Insensitive Prostatic Cancer*") OR ("Androgen Insensitive Prostatic Neoplasm*") OR ("Androgen Resistant Prostatic Cancer*") OR ("Androgen Resistant Prostatic Neoplasm*") OR ("castrate refractory metastatic PC*") OR ("castrate refractory metastatic prostate cancer") OR ("castrate refractory metastatic prostatic cancer") OR ("castrate resistant metastatic PC*") OR ("castrate resistant metastatic prostate cancer") OR ("castrate resistant metastatic prostatic cancer") OR ("castration refractory metastatic PC*") OR ("castration refractory metastatic prostate cancer") OR ("castration refractory metastatic prostatic cancer") OR ("castration resistant metastatic PC*") OR ("castration resistant metastatic prostate cancer") OR ("castration resistant metastatic prostatic cancer") OR ("castration resistant metastatic prostatic neoplasm") OR ("Castration Resistant Prostatic Cancer*") OR ("Castration Resistant Prostatic Neoplasm*") OR ("hormone refractory metastatic PC*") OR ("hormone refractory metastatic prostate cancer") OR ("hormone refractory metastatic prostatic cancer") OR ("Hormone Refractory Prostatic Cancer") OR ("Hormone Refractory Prostatic Neoplasms") OR ("hormone resistant metastatic PC*") OR ("hormone resistant metastatic prostate cancer") OR ("hormone resistant metastatic prostatic cancer") OR ("metastatic androgen independent PC*") OR ("metastatic androgen independent prostate cancer") OR ("metastatic androgen independent prostatic cancer") OR ("metastatic androgen insensitive prostate cancer") OR ("metastatic androgen insensitive prostatic cancer") OR ("metastatic castrate refractory PC*") OR ("metastatic castrate refractory prostate cancer") OR ("metastatic castrate refractory prostatic cancer") OR ("metastatic castrate resistant PC*") OR ("metastatic castrate resistant prostate cancer") OR ("metastatic castrate resistant prostatic cancer") OR ("metastatic castration refractory PC*") OR ("metastatic castration refractory prostate cancer") OR ("metastatic castration refractory prostatic cancer") OR ("metastatic castration resistant PC*") OR ("metastatic castration resistant prostate cancer") OR ("metastatic castration resistant prostatic cancer") OR ("metastatic castration resistant prostatic neoplasm") OR ("metastatic hormone refractory PC*") OR ("metastatic hormone refractory prostate cancer") OR ("metastatic hormone refractory prostatic cancer") OR ("metastatic hormone resistant PC*") OR ("metastatic hormone resistant prostate cancer") OR ("metastatic hormone resistant prostatic cancer"))) AND (TI=(("azd 2281") OR ("AZD221") OR ("azd2281") OR ("AZD-2281") OR ("ku 0059436") OR ("ku 59436") OR ("ku0059436") OR ("ku59436") OR ("lynparza") OR ("mk 7339") OR ("mk7339") OR ("ng 1002") OR ("ng1002") OR ("olaparib") OR ("ro 8508245") OR ("ro8508245")) OR AB=(("azd 2281") OR ("AZD221") OR ("azd2281") OR ("AZD-2281") OR ("ku 0059436") OR ("ku 59436") OR ("ku0059436") OR ("ku59436") OR ("lynparza") OR ("mk 7339") OR ("mk7339") OR ("ng 1002") OR ("ng1002") OR ("olaparib") OR ("ro 8508245") OR ("ro8508245")) OR AK=(("azd 2281") OR ("AZD221") OR ("azd2281") OR ("AZD-2281") OR ("ku 0059436") OR ("ku 59436") OR ("ku0059436") OR ("ku59436") OR ("lynparza") OR ("mk 7339") OR ("mk7339") OR ("ng 1002") OR ("ng1002") OR ("olaparib") OR ("ro 8508245") OR ("ro8508245"))) | 295 |

**Supplementary Table 2:** NIH Quality Assessment of Studies included.

| **NIH Quality Assessment of Controlled Intervention Studies** | | | | | | | | | | | | | | | | |
| --- | --- | --- | --- | --- | --- | --- | --- | --- | --- | --- | --- | --- | --- | --- | --- | --- |
| Question Number | 1 | 2 | 3 | 4 | 5 | 6 | 7 | 8 | 9 | 10 | 11 | 12 | 13 | 14 | Overall Score | Grade |
| ***de Bono 2020*** | *Y* | *Y* | *N* | *N* | *Y* | *Y* | *Y* | *Y* | *Y* | *Y* | *Y* | *Y* | *Y* | *Y* | 12 | Good |
| ***Clarke 2022*** | *Y* | *Y* | *Y* | *Y* | *Y* | *Y* | *Y* | *Y* | *Y* | *Y* | *Y* | *Y* | *Y* | *Y* | 14 | Good |
| ***Clarke 2018*** | *Y* | *Y* | *Y* | *Y* | *Y* | *Y* | *Y* | *Y* | *N* | *Y* | *Y* | *Y* | *Y* | *Y* | 13 | Good |
| ***Juaquin 2024*** | *Y* | *Y* | *N* | *N* | *Y* | *Y* | *Y* | *Y* | *Y* | *Y* | *Y* | *Y* | *Y* | *Y* | 12 | Good |
| ***Fred Saad 2023*** | *Y* | *Y* | *Y* | *Y* | *Y* | *Y* | *Y* | *Y* | *Y* | *Y* | *Y* | *Y* | *Y* | *Y* | 14 | Good |
| ***Emmanuel 2023*** | *Y* | *Y* | *N* | *N* | *Y* | *Y* | *Y* | *Y* | *Y* | *Y* | *Y* | *Y* | *Y* | *Y* | 12 | Good |
| ***Kim 2022*** | *Y* | *Y* | *N* | *N* | *N* | *Y* | *Y* | *Y* | *Y* | *Y* | *Y* | *Y* | *Y* | *Y* | 11 | Good |
| **NIH Quality Assessment Tool for Observational Cohort and Cross-Sectional Studies** | | | | | | | | | | | | | | | | |
| Question Number | 1 | 2 | 3 | 4 | 5 | 6 | 7 | 8 | 9 | 10 | 11 | 12 | 13 | 14 | Overall Score | Grade |
| ***JUN XIE2024*** | *Y* | *Y* | *Y* | *Y* | *N* | *Y* | *Y* | *N* | *Y* | *N* | *Y* | *N* | *Y* | *Y* | 10 | Fair |

**Supplementary Table 3:** League table in different gene-mutated subgroups.

| **PFS** | | | | | |
| --- | --- | --- | --- | --- | --- |
| **ALL** | **Olaparib** | 0.46 (0.25, 0.83) | 0.62 (0.39, 0.97) | 0.3 (0.17, 0.53) | 0.46 (0.25, 0.88) |
|  | 2.2 (1.21, 4) | **NHA** | 1.36 (0.64, 2.86) | 0.66 (0.55, 0.79) | 1.02 (0.83, 1.26) |
|  | 1.62 (1.03, 2.55) | 0.74 (0.35, 1.56) | **Olaparib + Cediranib** | 0.48 (0.23, 1.01) | 0.75 (0.35, 1.64) |
|  | 3.34 (1.89, 5.9) | 1.52 (1.27, 1.82) | 2.06 (0.99, 4.26) | **Olaparib + Abiraterone** | 1.55 (1.17, 2.04) |
|  | 2.15 (1.14, 4.06) | 0.98 (0.8, 1.21) | 1.33 (0.61, 2.9) | 0.64 (0.49, 0.85) | **Olaparib + Pembrolizumab** |
| **HRRmt** | **Olaparib** | 2.04 (1.58, 2.63) | 0.65 (0.27, 1.52) | 0.97 (0.63, 1.49) | 1.41 (0.88, 2.28) |
|  | 0.49 (0.38, 0.63) | **NHA** | 0.32 (0.13, 0.78) | 0.48 (0.34, 0.67) | 0.69 (0.46, 1.04) |
|  | 1.54 (0.66, 3.65) | 3.14 (1.29, 7.76) | **Olaparib + Cediranib** | 1.5 (0.58, 3.9) | 2.16 (0.82, 5.82) |
|  | 1.03 (0.67, 1.59) | 2.1 (1.49, 2.98) | 0.67 (0.26, 1.74) | **Olaparib + Abiraterone** | 1.45 (0.85, 2.47) |
|  | 0.71 (0.44, 1.14) | 1.45 (0.97, 2.17) | 0.46 (0.17, 1.23) | 0.69 (0.4, 1.17) | **Olaparib + Pembrolizumab** |
| **BRCAmt** | **Olaparib** | 3.52 (2.51, 4.93) | 0.98 (0.32, 2.99) | 0.61 (0.41, 0.91) | 1.41 (0.67, 2.97) |
|  | 0.28 (0.2, 0.4) | **NHA** | 0.28 (0.09, 0.89) | 0.17 (0.12, 0.26) | 0.4 (0.21, 0.78) |
|  | 1.02 (0.33, 3.11) | 3.59 (1.12, 11.41) | **Olaparib + Cediranib** | 0.62 (0.19, 2.03) | 1.43 (0.37, 5.45) |
|  | 1.64 (1.1, 2.47) | 5.78 (3.86, 8.64) | 1.61 (0.49, 5.29) | **Olaparib + Abiraterone** | 2.31 (1.06, 5.01) |
|  | 0.71 (0.34, 1.49) | 2.5 (1.28, 4.87) | 0.7 (0.18, 2.69) | 0.43 (0.2, 0.94) | **Olaparib + Pembrolizumab** |
| **HRRwt** | **NHA** | 0.7 (0.55, 0.89) | 1.26 (0.97, 1.63) |  |  |
|  | 1.43 (1.12, 1.82) | **Olaparib + Abiraterone** | 1.81 (1.26, 2.57) |  |  |
|  | 0.79 (0.61, 1.03) | 0.55 (0.39, 0.79) | **Olaparib + Pembrolizumab** |  |  |
| **BRCAmt** | **NHA** | 0.85 (0.66, 1.1) | 1.2 (0.95, 1.51) |  |  |
|  | 1.17 (0.91, 1.51) | **Olaparib + Abiraterone** | 1.41 (1, 1.98) |  |  |
|  | 0.83 (0.66, 1.05) | 0.71 (0.51, 1) | **Olaparib + Pembrolizumab** |  |  |
| **OS** |  |  |  |  |  |
| **ALL** | **Olaparib** | 0.3 (0.13, 0.73) | 1.3 (0.7, 2.39) | 0.25 (0.11, 0.59) | 0.28 (0.12, 0.69) |
|  | 3.3 (1.38, 7.86) | **NHA** | 4.28 (1.48, 12.34) | 0.83 (0.69, 0.99) | 0.94 (0.77, 1.15) |
|  | 0.77 (0.42, 1.43) | 0.23 (0.08, 0.68) | **Olaparib + Cediranib** | 0.19 (0.07, 0.55) | 0.22 (0.08, 0.65) |
|  | 3.97 (1.7, 9.27) | 1.21 (1.01, 1.45) | 5.18 (1.81, 14.67) | **Olaparib + Abiraterone** | 1.14 (0.87, 1.48) |
|  | 3.51 (1.44, 8.5) | 1.06 (0.87, 1.29) | 4.55 (1.55, 13.31) | 0.88 (0.67, 1.15) | **Olaparib + Pembrolizumab** |
| **HRRmt** | **Olaparib** | 1.49 (1.08, 2.05) | 0.98 (0.6, 1.61) | 1.31 (0.78, 2.2) |  |
|  | 0.67 (0.49, 0.93) | **NHA** | 0.66 (0.46, 0.96) | 0.88 (0.58, 1.32) |  |
|  | 1.02 (0.62, 1.66) | 1.52 (1.04, 2.19) | **Olaparib + Cediranib** | 1.33 (0.77, 2.31) |  |
|  | 0.76 (0.45, 1.28) | 1.14 (0.76, 1.71) | 0.75 (0.43, 1.31) | **Olaparib + Abiraterone** |  |
| **BRCAmt** | **Olaparib** | 1.54 (1.04, 2.28) | 0.41 (0.21, 0.8) | 0.8 (0.38, 1.71) |  |
|  | 0.65 (0.44, 0.96) | **NHA** | 0.27 (0.15, 0.49) | 0.52 (0.27, 1) |  |
|  | 2.42 (1.24, 4.69) | 3.74 (2.04, 6.84) | **Olaparib + Cediranib** | 1.94 (0.8, 4.75) |  |
|  | 1.25 (0.59, 2.65) | 1.93 (1, 3.68) | 0.51 (0.21, 1.25) | **Olaparib + Abiraterone** |  |
| **HRRwt** | **NHA** | 0.89 (0.7, 1.14) | 0.97 (0.76, 1.23) |  |  |
|  | 1.12 (0.88, 1.43) | **Olaparib + Abiraterone** | 1.09 (0.77, 1.53) |  |  |
|  | 1.03 (0.81, 1.31) | 0.92 (0.65, 1.29) | **Olaparib + Pembrolizumab** |  |  |
| **BRCAmt** | **NHA** | 1.13 (0.87, 1.47) | 1 (0.8, 1.25) |  |  |
|  | 0.88 (0.68, 1.15) | **Olaparib + Abiraterone** | 0.88 (0.63, 1.25) |  |  |
|  | 1 (0.8, 1.25) | 1.13 (0.8, 1.59) | **Olaparib + Pembrolizumab** |  |  |
| **AE&SAE** |  |  |  |  |  |
| **AE** | **Olaparib** | 0.98 (0.76, 1.22) | 1.02 (0.9, 1.16) | 1 (0.78, 1.25) | 1.7 (1.29, 2.2) |
|  | 1.02 (0.82, 1.32) | **NHA** | 1.05 (0.81, 1.38) | 1.02 (1, 1.05) | 1.74 (1.55, 1.99) |
|  | 0.98 (0.86, 1.11) | 0.96 (0.72, 1.23) | **Olaparib + Cediranib** | 0.98 (0.74, 1.26) | 1.67 (1.23, 2.21) |
|  | 1 (0.8, 1.29) | 0.98 (0.95, 1) | 1.02 (0.8, 1.35) | **Olaparib + Abiraterone** | 1.7 (1.51, 1.94) |
|  | 0.59 (0.45, 0.78) | 0.57 (0.5, 0.65) | 0.6 (0.45, 0.81) | 0.59 (0.51, 0.66) | **Olaparib + Pembrolizumab** |
| **SAE** | **Olaparib** | 0.74 (0.26, 1.91) | 3.55 (1.89, 7.81) | 1 (0.36, 2.55) | 2.89 (0.96, 8.23) |
|  | 1.35 (0.52, 3.84) | **NHA** | 4.92 (1.54, 17.61) | 1.35 (1.18, 1.54) | 3.9 (2.65, 6.07) |
|  | 0.28 (0.13, 0.53) | 0.2 (0.06, 0.65) | **Olaparib + Cediranib** | 0.27 (0.08, 0.87) | 0.8 (0.21, 2.76) |
|  | 1 (0.39, 2.81) | 0.74 (0.65, 0.85) | 3.65 (1.15, 12.93) | **Olaparib + Abiraterone** | 2.9 (1.92, 4.6) |
|  | 0.35 (0.12, 1.04) | 0.26 (0.16, 0.38) | 1.26 (0.36, 4.74) | 0.35 (0.22, 0.52) | **Olaparib + Pembrolizumab** |

**Supplementary Table 4:** League table in PSA subgroups.

| **PSA＜100 subgroup** | | | | |
| --- | --- | --- | --- | --- |
| **Olaparib**  **PFS** | 0.58 (0.26, 1.32) | 0.37 (0.17, 0.82) |  |  |
| 1.72 (0.76, 3.91) 2.71 (1.22, 6.03) | **NHA**  1.57 (1.32, 1.88) | 0.64 (0.53, 0.76)  **Olaparib + Abiraterone** |  |  |
| **PSA＞100 subgroup** |  |  |  |  |
| **Olaparib**  **OS** | 1.49 (1.08, 2.06) | 1.3 (0.7, 2.39) | 0.25 (0.11, 0.59) | 1.4 (0.96, 2.05) |
| 0.67 (0.49, 0.92) | **NHA** | 0.87 (0.43, 1.73) | 0.17 (0.07, 0.42) | 0.94 (0.77, 1.14) |
| 0.77 (0.42, 1.42) | 1.15 (0.58, 2.3) | **Olaparib + Cediranib** | 0.19 (0.07, 0.55) | 1.08 (0.53, 2.21) |
| 3.99 (1.7, 9.35) | 5.96 (2.4, 14.85) | 5.18 (1.82, 14.8) | **Olaparib + Abiraterone** | 5.6 (2.2, 14.27) |
| 0.71 (0.49, 1.04) | 1.06 (0.87, 1.3) | 0.93 (0.45, 1.89) | 0.18 (0.07, 0.46) | **Olaparib + Pembrolizumab** |
| **Olaparib**  **PFS** | 2.04 (1.58, 2.63) | 0.62 (0.39, 0.97) | 0.3 (0.17, 0.53) | 2.08 (1.5, 2.89) |
| 0.49 (0.38, 0.63) | **NHA** | 0.3 (0.18, 0.51) | 0.15 (0.08, 0.27) | 1.02 (0.83, 1.26) |
| 1.62 (1.03, 2.55) | 3.31 (1.97, 5.55) | **Olaparib + Cediranib** | 0.49 (0.23, 1.01) | 3.38 (1.92, 5.9) |
| 3.33 (1.89, 5.9) | 6.81 (3.66, 12.71) | 2.05 (0.99, 4.27) | **Olaparib + Abiraterone** | 6.94 (3.6, 13.4) |
| 0.48 (0.35, 0.67) | 0.98 (0.79, 1.21) | 0.3 (0.17, 0.52) | 0.14 (0.07, 0.28) | **Olaparib + Pembrolizumab** |

**Supplementary Table 5:** PFS&OS Sensitivity analysis (after removal of non-prospective studies)

| **PFS** | | | | **Hazard Ratio** | **SUCRA** | |  |  |  |
| --- | --- | --- | --- | --- | --- | --- | --- | --- | --- |
|  |  |  |  | **(95% CrI)** | **(%)** | |  |  |  |
| **All Patients** | | | | | | |  |  |  |
|  | Reference:Olaparib + Abiraterone | | | 1 | 99.95% | |  |  |  |
|  | NHA | | | 1.50 (1.30, 1.80) | 28.77% | |  |  |  |
|  | Olaparib + Pembrolizumab | | | 1.60 (1.20, 2.00) | 21.29% | |  |  |  |
| **HRR-muted Subgroup** | | | | | | |  |  |  |
|  | Reference：Olaparib | | | 1 | 63.25% | |  |  |  |
|  | NHA | | | 2.00 (1.60, 2.60) | 1.03% | |  |  |  |
|  | Olaparib + Cediranib | | | 0.65 (0.28, 1.50) | 89.17% | |  |  |  |
|  | Olaparib + Abiraterone | | | 0.97 (0.63, 1.50) | 66.88% | |  |  |  |
|  | Olaparib + Pembrolizumab | | | 1.40 (0.88, 2.30) | 29.68% | |  |  |  |
| **BRCA-muted Subgroup** | | | | | | |  |  |  |
|  | Reference：Olaparib | | | 1 | 62.99% | |  |  |  |
|  | NHA | | | 3.80 (2.70, 5.50) | 0.35% | |  |  |  |
|  | Olaparib + Cediranib | | | 0.98 (0.32, 3.00) | 64.93% | |  |  |  |
|  | Olaparib + Abiraterone | | | 0.78 (0.47, 1.30) | 85.87% | |  |  |  |
|  | Olaparib + Pembrolizumab | | | 1.50 (0.73, 3.30) | 35.86% | |  |  |  |
| **nonHRR-muted Subgroup** | | | | | | |  |  |  |
|  | Reference:Olaparib + Abiraterone | | | 1 | 48.15% | |  |  |  |
|  | NHA | | | 1.40 (1.10-1.80) | 99.89% | |  |  |  |
|  | Olaparib + Pembrolizumab | | | 1.80 (1.30-2.60) | 1.96% | |  |  |  |
| **nonBRCA-muted Subgroup** | | | | | | |  |  |  |
|  | Reference:Olaparib + Abiraterone | | | 1 | 52.12% | |  |  |  |
|  | NHA | | | 1.20 (0.91, 1.5) | 93.57% | |  |  |  |
|  | Olaparib + Pembrolizumab | | | 1.40 (1.0, 2.00) | 4.31% | |  |  |  |
| **PFS** | |  |  | | |  | |  | |
|  |  |  |  | | |  | |  | |
| ALL | | | | | | | | | |
| **NHA** | | 0.66 (0.55, 0.79) | 1.02 (0.83, 1.26) | | |  | |  | |
| 1.52 (1.27, 1.82) | | **Olaparib + Abiraterone** | 1.55 (1.18, 2.05) | | |  | |  | |
| 0.98 (0.79, 1.21) | | 0.64 (0.49, 0.85) | **Olaparib + Pembrolizumab** | | |  | |  | |
| HRRm | | | | | | | | | |
| **Olaparib** | | 2.04 (1.58, 2.63) | 0.65 (0.28, 1.52) | | | 0.97 (0.63, 1.49) | | 1.41 (0.88, 2.28) | |
| 0.49 (0.38, 0.63) | | **NHA** | 0.32 (0.13, 0.77) | | | 0.48 (0.34, 0.67) | | 0.69 (0.46, 1.03) | |
| 1.54 (0.66, 3.62) | | 3.15 (1.29, 7.67) | **Olaparib + Cediranib** | | | 1.49 (0.58, 3.89) | | 2.17 (0.82, 5.79) | |
| 1.03 (0.67, 1.58) | | 2.1 (1.49, 2.98) | 0.67 (0.26, 1.73) | | | **Olaparib + Abiraterone** | | 1.45 (0.85, 2.47) | |
| 0.71 (0.44, 1.14) | | 1.45 (0.97, 2.16) | 0.46 (0.17, 1.21) | | | 0.69 (0.41, 1.17) | | **Olaparib + Pembrolizumab** | |
| BRCAm | | | | | | | | | |
| **Olaparib** | | 3.84 (2.71, 5.47) | 0.98 (0.32, 3) | | | 0.78 (0.47, 1.28) | | 1.54 (0.73, 3.26) | |
| 0.26 (0.18, 0.37) | | **NHA** | 0.26 (0.08, 0.83) | | | 0.2 (0.13, 0.32) | | 0.4 (0.21, 0.77) | |
| 1.02 (0.33, 3.11) | | 3.91 (1.21, 12.61) | **Olaparib + Cediranib** | | | 0.79 (0.23, 2.69) | | 1.56 (0.41, 6) | |
| 1.28 (0.78, 2.11) | | 4.93 (3.17, 7.67) | 1.26 (0.37, 4.28) | | | **Olaparib + Abiraterone** | | 1.97 (0.89, 4.36) | |
| 0.65 (0.31, 1.38) | | 2.5 (1.29, 4.85) | 0.64 (0.17, 2.44) | | | 0.51 (0.23, 1.12) | | **Olaparib + Pembrolizumab** | |
| nonHRRm | | | | | | | | | |
| **NHA** | | 0.7 (0.55, 0.89) | 1.26 (0.97, 1.63) | | |  | |  | |
| 1.43 (1.13, 1.82) | | **Olaparib + Abiraterone** | 1.8 (1.27, 2.57) | | |  | |  | |
| 0.79 (0.61, 1.03) | | 0.55 (0.39, 0.79) | **Olaparib + Pembrolizumab** | | |  | |  | |
| nonBRCAm | | | | | | | | | |
| **NHA** | | 0.85 (0.66, 1.1) | 1.2 (0.95, 1.51) | | |  | |  | |
| 1.17 (0.91, 1.51) | | **Olaparib + Abiraterone** | 1.41 (1, 1.98) | | |  | |  | |
| 0.83 (0.66, 1.05) | | 0.71 (0.51, 1) | **Olaparib + Pembrolizumab** | | |  | |  | |
| **OS** | | | | **Hazard Ratio** | **SUCRA** | |  |  |  |
|  |  |  |  | **(95% CrI)** |  |  |  |  |  |
| **All Patients** | | | | | | |  |  |  |
|  | Reference：Olaparib + Abiraterone | | | 1 | 90.29% | |  |  |  |
|  | NHA | | | 1.20 (1.00, 1.40) | 14.46% | |  |  |  |
|  | Olaparib + Pembrolizumab | | | 1.10 (0.87, 1.50) | 45.26% | |  |  |  |
| **HRR-muted Subgroup** | | | | | | |  |  |  |
|  | Reference：Olaparib | | | 1 | 77.19% | |  |  |  |
|  | NHA | | | 1.50 (1.10-2.10) | 9.66% | |  |  |  |
|  | Olaparib + Abiraterone | | | 0.98 (0.60-1.60) | 78.60% | |  |  |  |
|  | Olaparib + Pembrolizumab | | | 1.30 (0.78, 2.20) | 34.55% | |  |  |  |
| **BRCA-muted Subgroup** | | | | | | |  |  |  |
|  | Reference：Olaparib | | | 1 | 44.28% | |  |  |  |
|  | NHA | | | 1.60 (1.10, 2.40) | 1.26% | |  |  |  |
|  | Olaparib + Abiraterone | | | 0.46 (0.21, 1.00) | 95.22% | |  |  |  |
|  | Olaparib + Pembrolizumab | | | 0.83 (0.38, 1.80) | 59.24% | |  |  |  |
| **nonHRR-muted Subgroup** | | | | | | |  |  |  |
|  | Reference:Olaparib + Abiraterone | | | 1 | 29.97% | |  |  |  |
|  | NHA | | | 1.10 (0.88-1.40) | 75.66% | |  |  |  |
|  | Olaparib + Pembrolizumab | | | 1.10 (0.78-1.50) | 45.47% | |  |  |  |
| **nonBRCA-muted Subgroup** | | | | | | |  |  |  |
|  | Reference:Olaparib + Abiraterone | | | 1 | 65.88% | |  |  |  |
|  | NHA | | | 0.89 (0.68-1.10) | 21.05% | |  |  |  |
|  | Olaparib + Pembrolizumab | | | 0.88 (0.63-1.20) | 63.07% | |  |  |  |
| **OS** | | |  | | |  | | |  |
|  |  |  |  | | |  | | |  |
| **All Patients** | | | | | | | | |  |
| **Olaparib + Abiraterone** | 1.21 (1.01, 1.45) | | 1.14 (0.87, 1.48) | | |  | | |  |
| 0.83 (0.69, 0.99) | **NHA** | | 0.94 (0.77, 1.15) | | |  | | |  |
| 0.88 (0.67, 1.15) | 1.06 (0.87, 1.3) | | **Olaparib + Pembrolizumab** | | |  | | |  |
| **HRR-muted Subgroup** | | | | | | | | |  |
| **Olaparib** | 1.49 (1.08, 2.05) | | 0.98 (0.6, 1.61) | | | 1.31 (0.78, 2.2) | | |  |
| 0.67 (0.49, 0.93) | **NHA** | | 0.66 (0.45, 0.96) | | | 0.88 (0.59, 1.32) | | |  |
| 1.02 (0.62, 1.66) | 1.52 (1.04, 2.2) | | **Olaparib + Abiraterone** | | | 1.33 (0.77, 2.32) | | |  |
| 0.76 (0.45, 1.28) | 1.14 (0.76, 1.7) | | 0.75 (0.43, 1.3) | | | **Olaparib + Pembrolizumab** | | |  |
| **BRCA-muted Subgroup** | | | | | | | | |  |
| **Olaparib** | 1.59 (1.06, 2.39) | | 0.46 (0.21, 1.03) | | | 0.83 (0.38, 1.78) | | |  |
| 0.63 (0.42, 0.95) | **NHA** | | 0.29 (0.15, 0.58) | | | 0.52 (0.27, 1) | | |  |
| 2.17 (0.97, 4.85) | 3.45 (1.72, 6.9) | | **Olaparib + Abiraterone** | | | 1.8 (0.69, 4.61) | | |  |
| 1.21 (0.56, 2.61) | 1.92 (1, 3.69) | | 0.56 (0.22, 1.44) | | | **Olaparib + Pembrolizumab** | | |  |
| **nonHRR-muted Subgroup** | | | | | | | | |  |
| **Olaparib + Abiraterone** | 0.89 (0.7, 1.14) | | 0.97 (0.76, 1.23) | | |  | | |  |
| 1.12 (0.88, 1.43) | **NHA** | | 1.09 (0.77, 1.53) | | |  | | |  |
| 1.03 (0.81, 1.31) | 0.92 (0.65, 1.3) | | **Olaparib + Pembrolizumab** | | |  | | |  |
| **nonBRCA-muted Subgroup** | | | | | | | | |  |
| **Olaparib + Abiraterone** | 1.13 (0.87, 1.47) | | 1 (0.8, 1.25) | | |  | | |  |
| 0.89 (0.68, 1.15) | **NHA** | | 0.89 (0.63, 1.25) | | |  | | |  |
| 1 (0.8, 1.25) | 1.13 (0.8, 1.59) | | **Olaparib + Pembrolizumab** | | |  | | |  |

**Supplementary Table 6:** Frequentist Network Meta-Analysis Plot (with emphasis on Tau-squared)


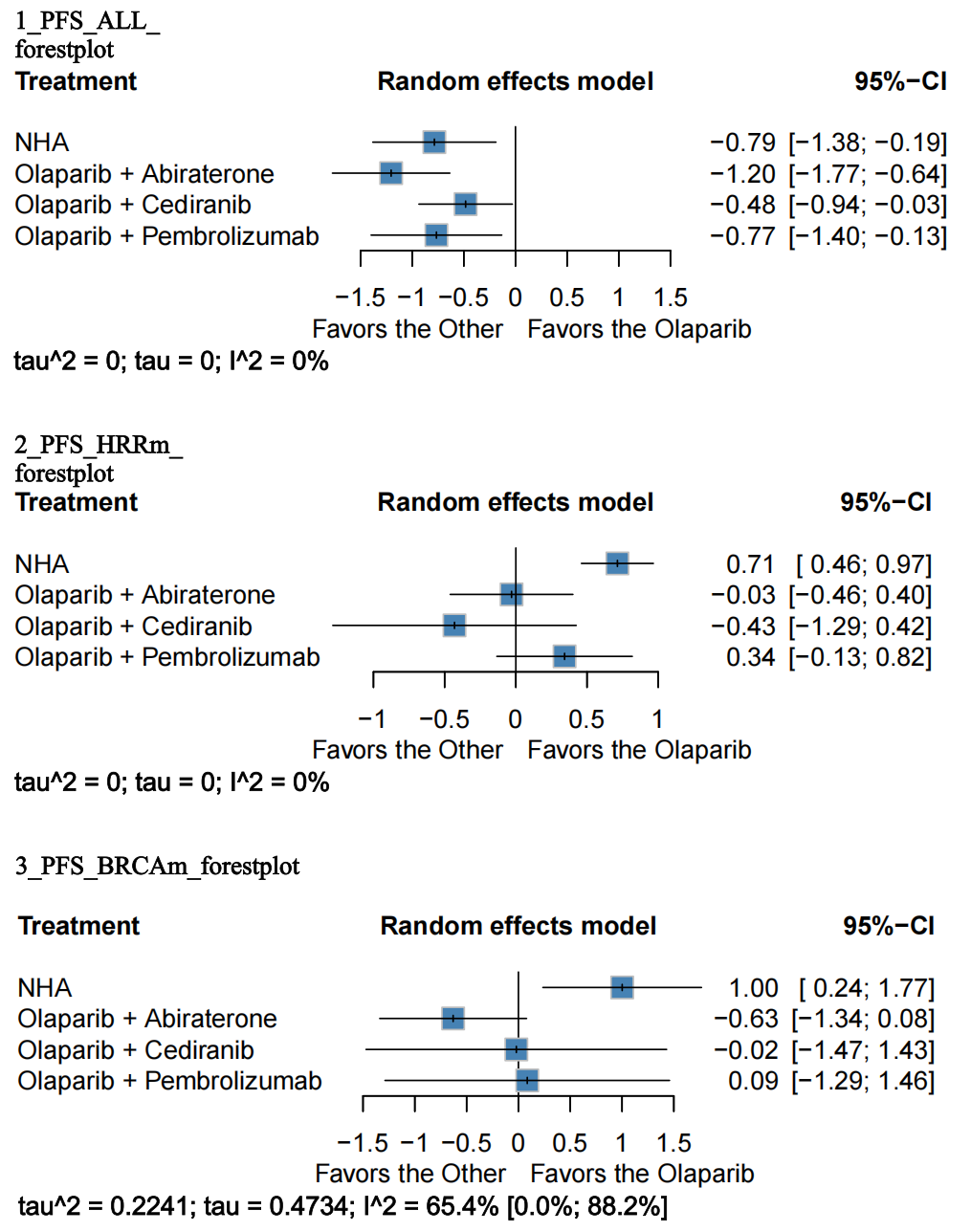


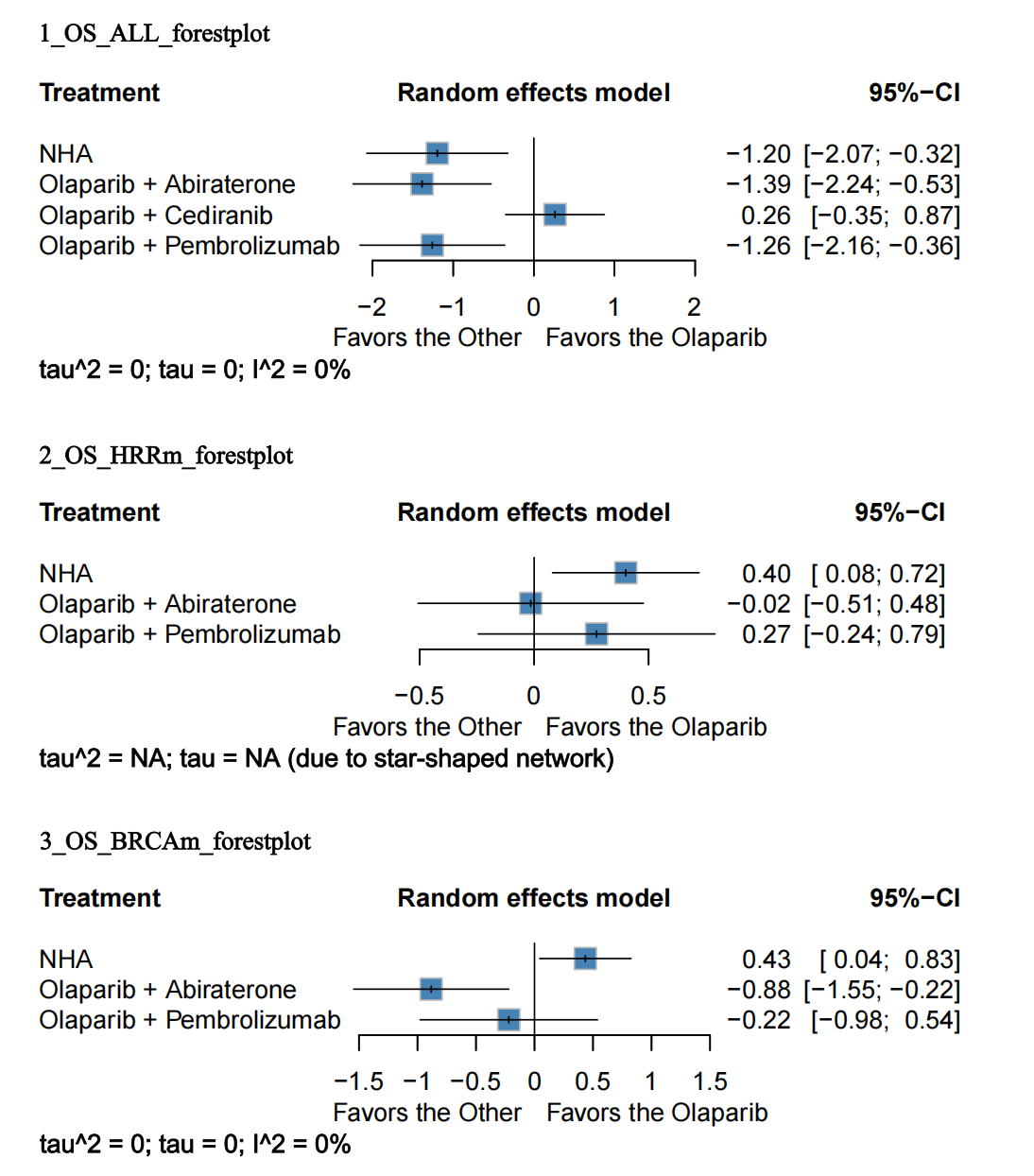


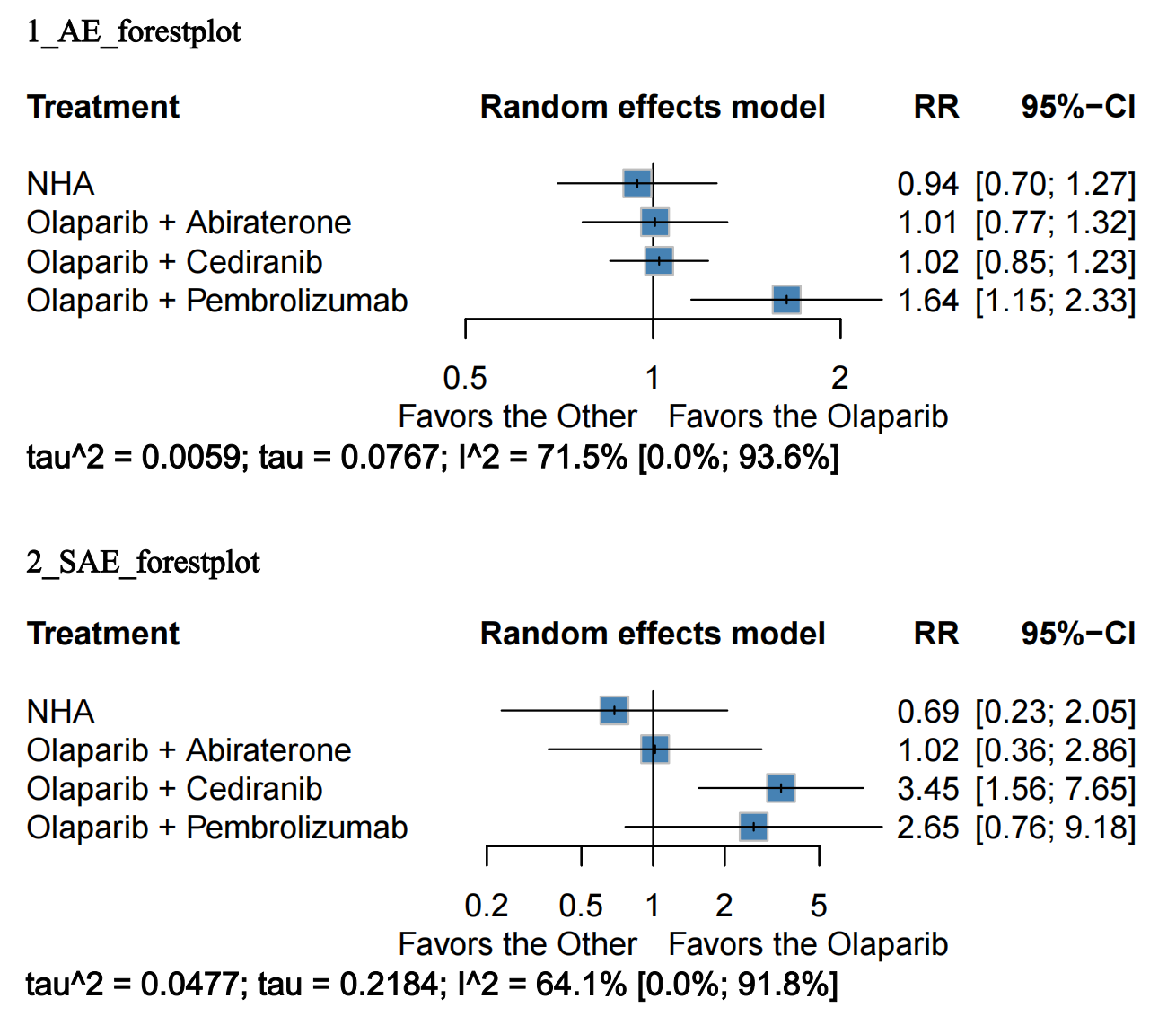

Supplement: Supplementary Data 1 [file mmc1.docx]
