# Supplementary material for: PARP Inhibitor Olaparib and Its Combination Therapy in Metastatic Castration-resistant Prostate Cancer: A Systematic Review and Network Meta-analysis
Source: Eur Urol Open Sci. 2025 Dec 31;84:1–12. doi: 10.1016/j.euros.2025.12.014 (PMC12804617; doi:10.1016/j.euros.2025.12.014)
Supplement: Supplementary Data 2 [file mmc2.docx]

**
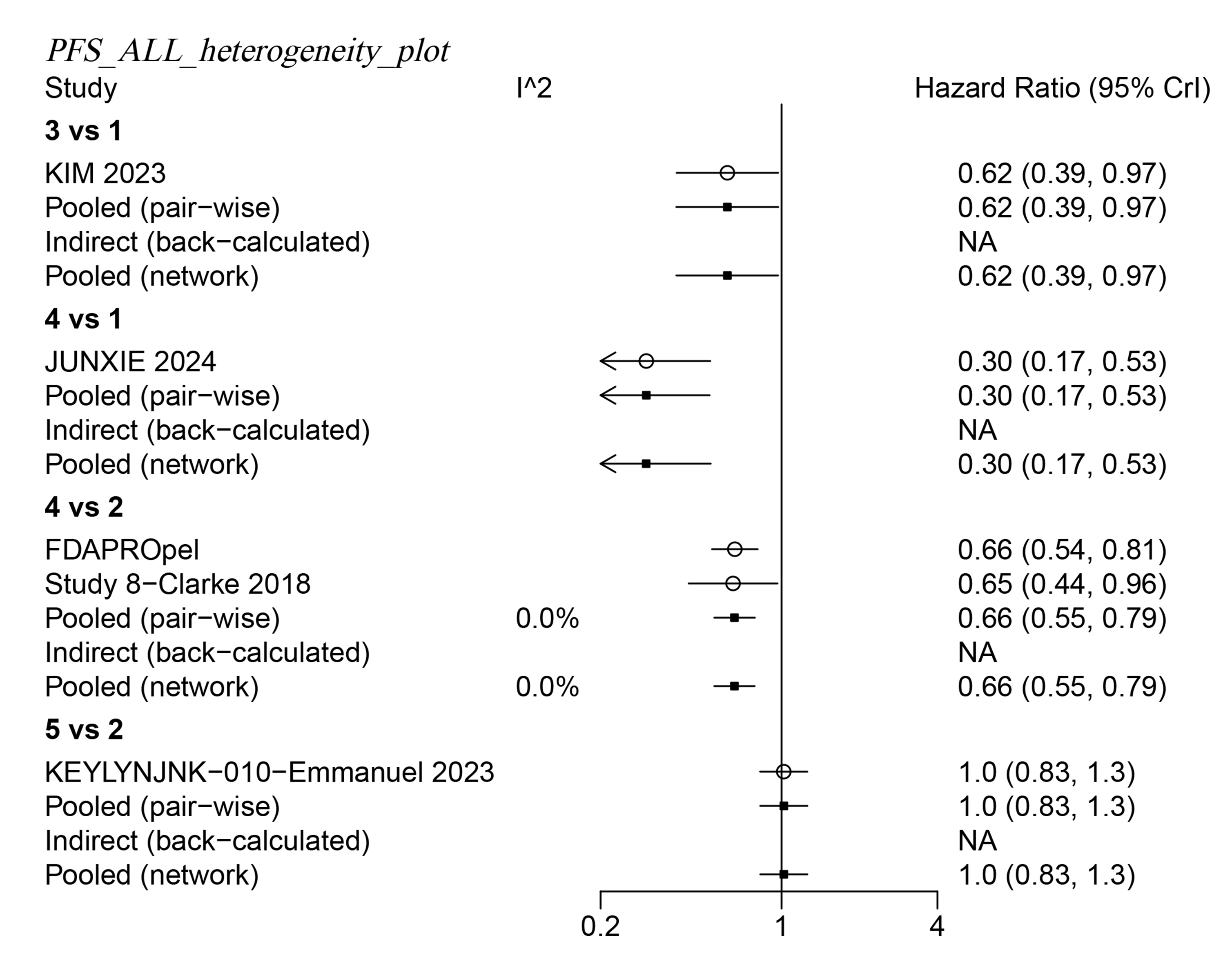
**

**Supplementary Figure 1** PFS ALL heterogeneity plot.


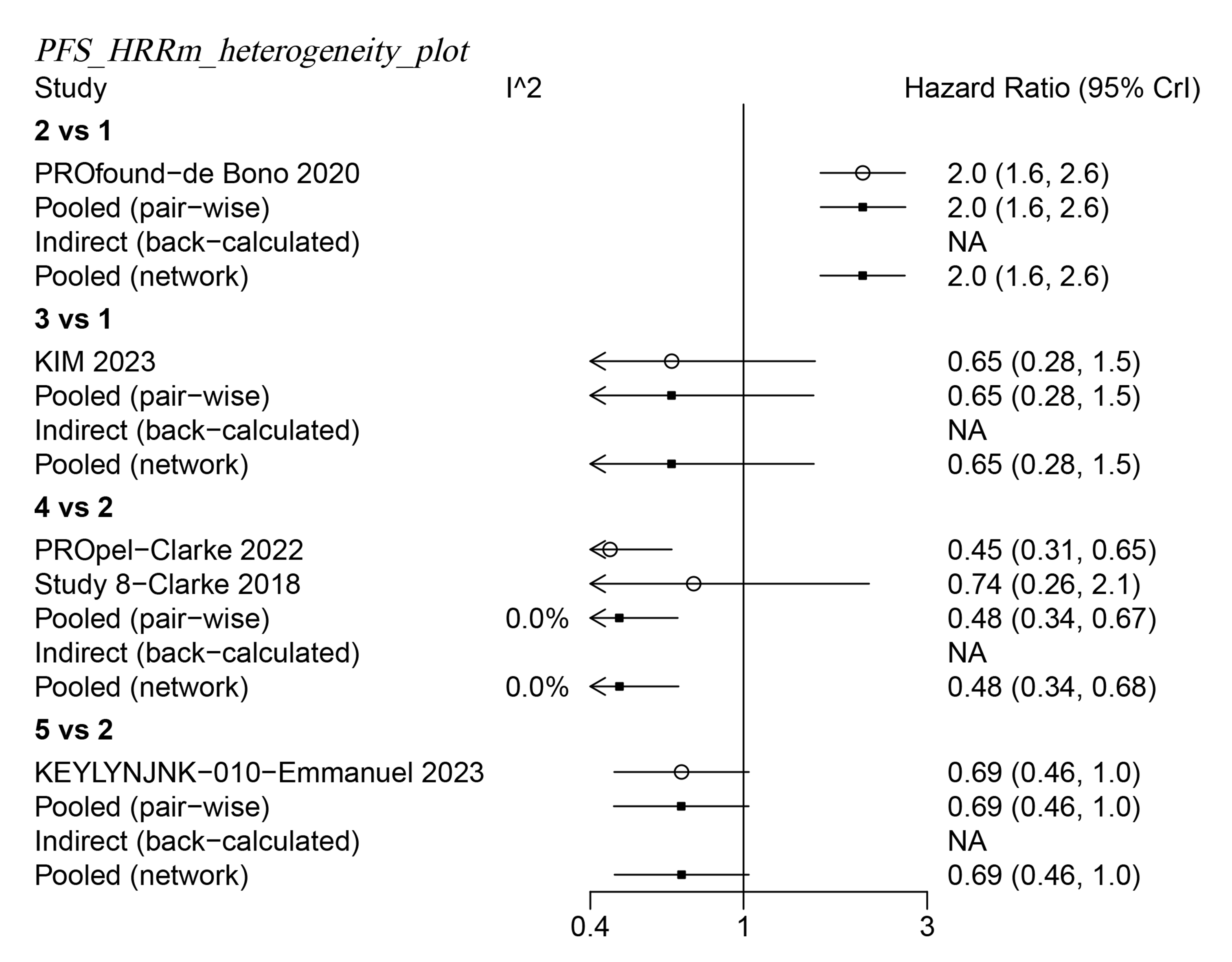


**Supplementary Figure 2** PFS HRRm heterogeneity plot.


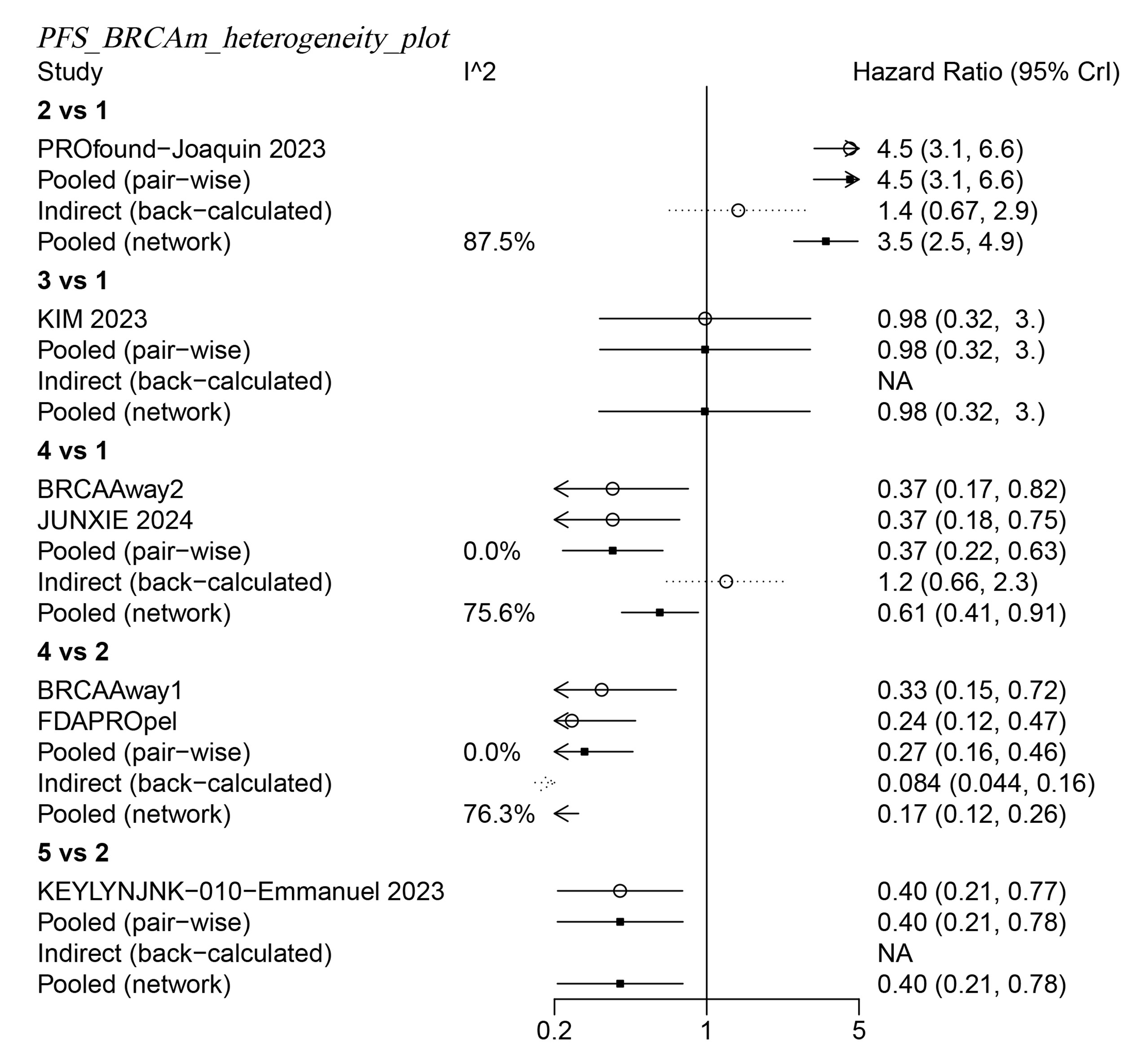


**Supplementary Figure 3** PFS BRCAm heterogeneity plot.


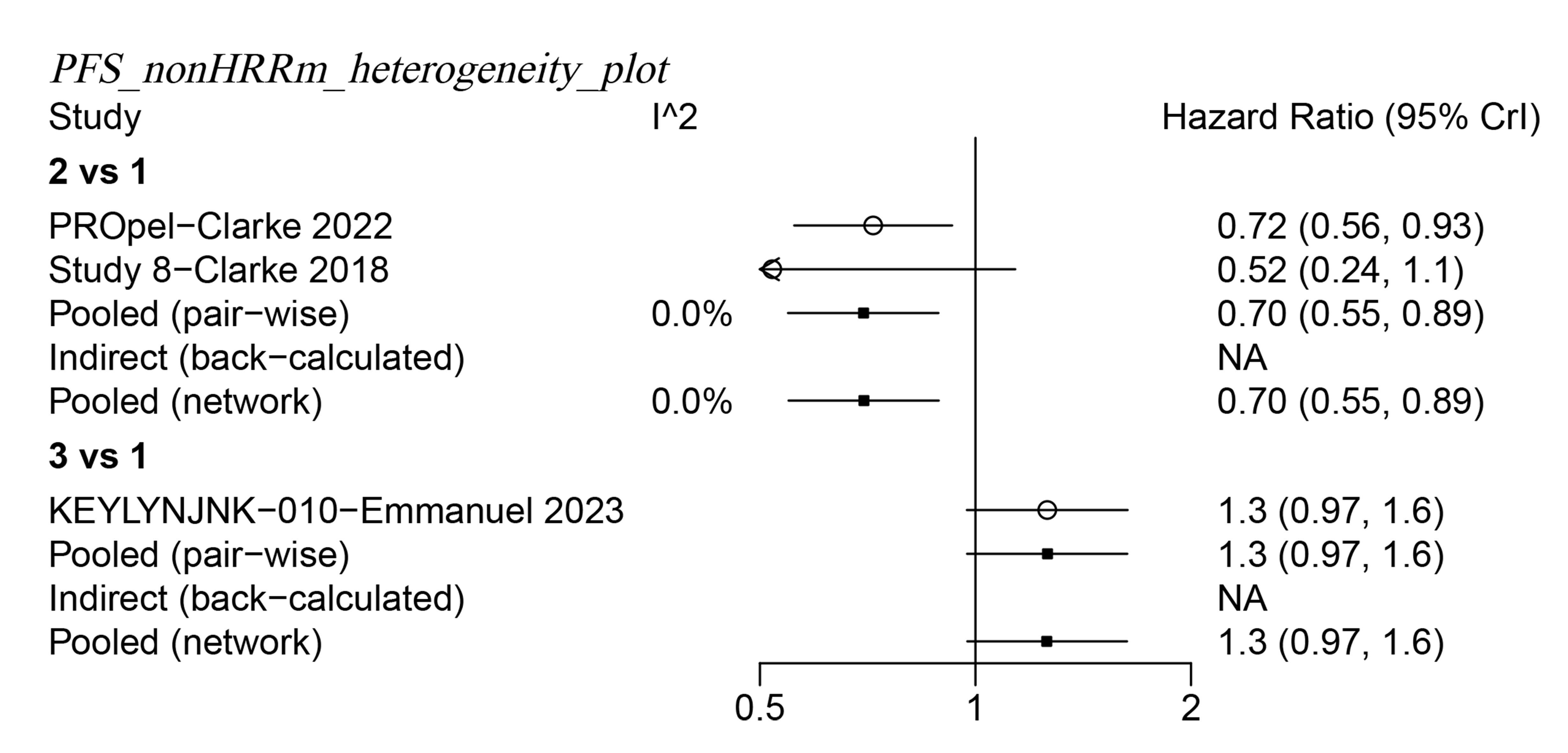


**Supplementary Figure 4** PFS nonHRRm heterogeneity plot.


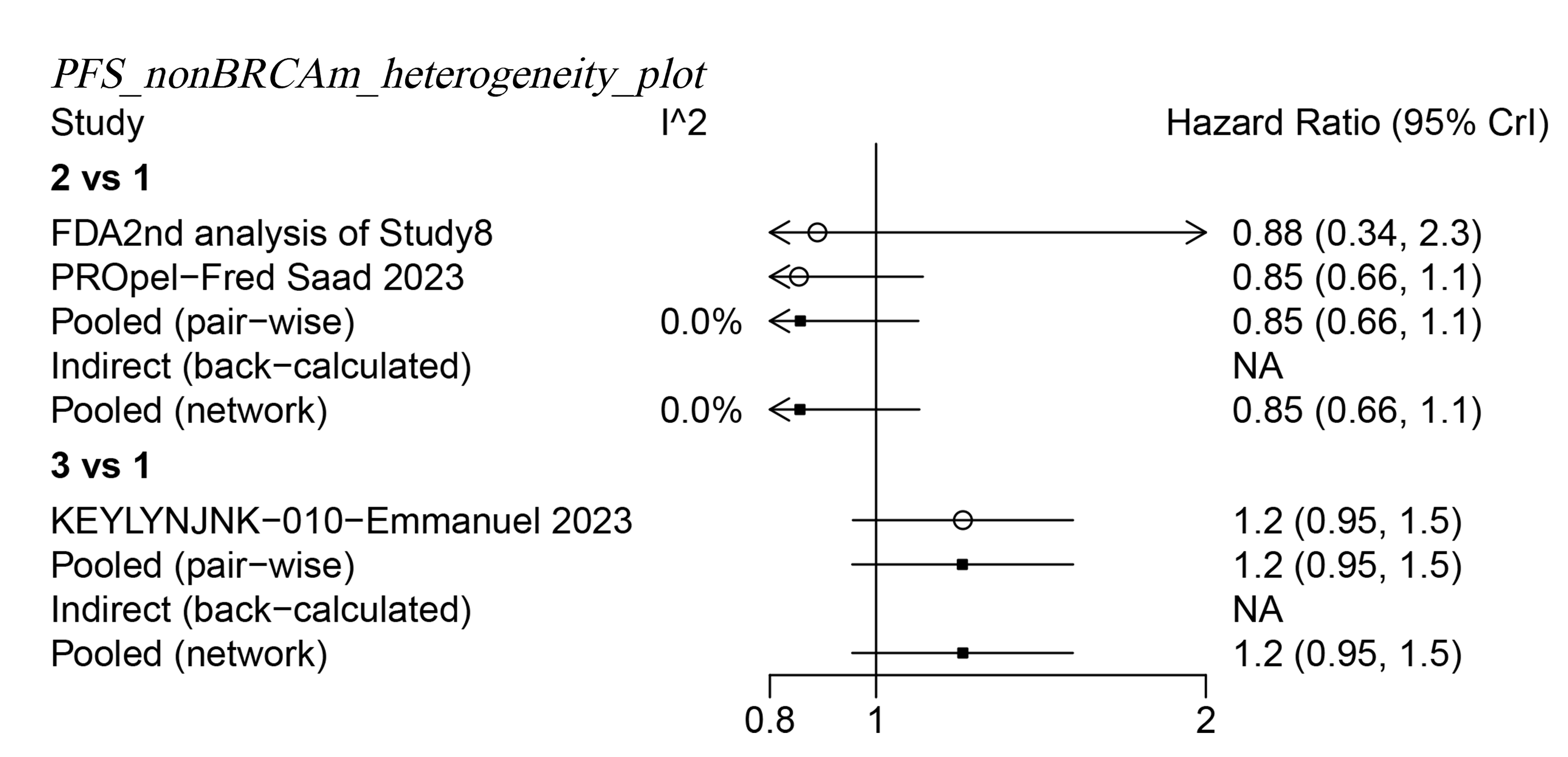


**Supplementary Figure 5** PFS nonBRCAm heterogeneity plot.


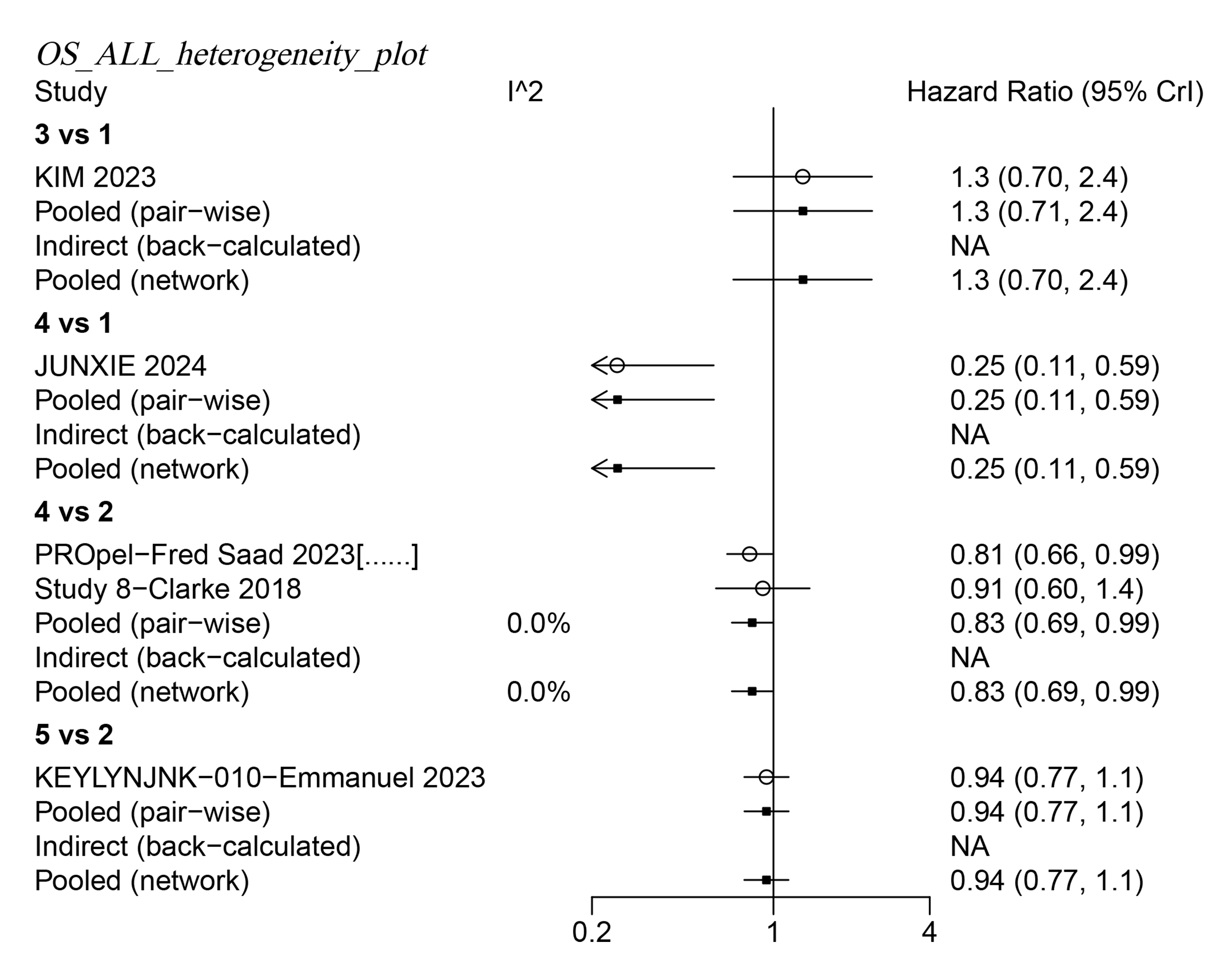


**Supplementary Figure 6** OS ALL heterogeneity plot.


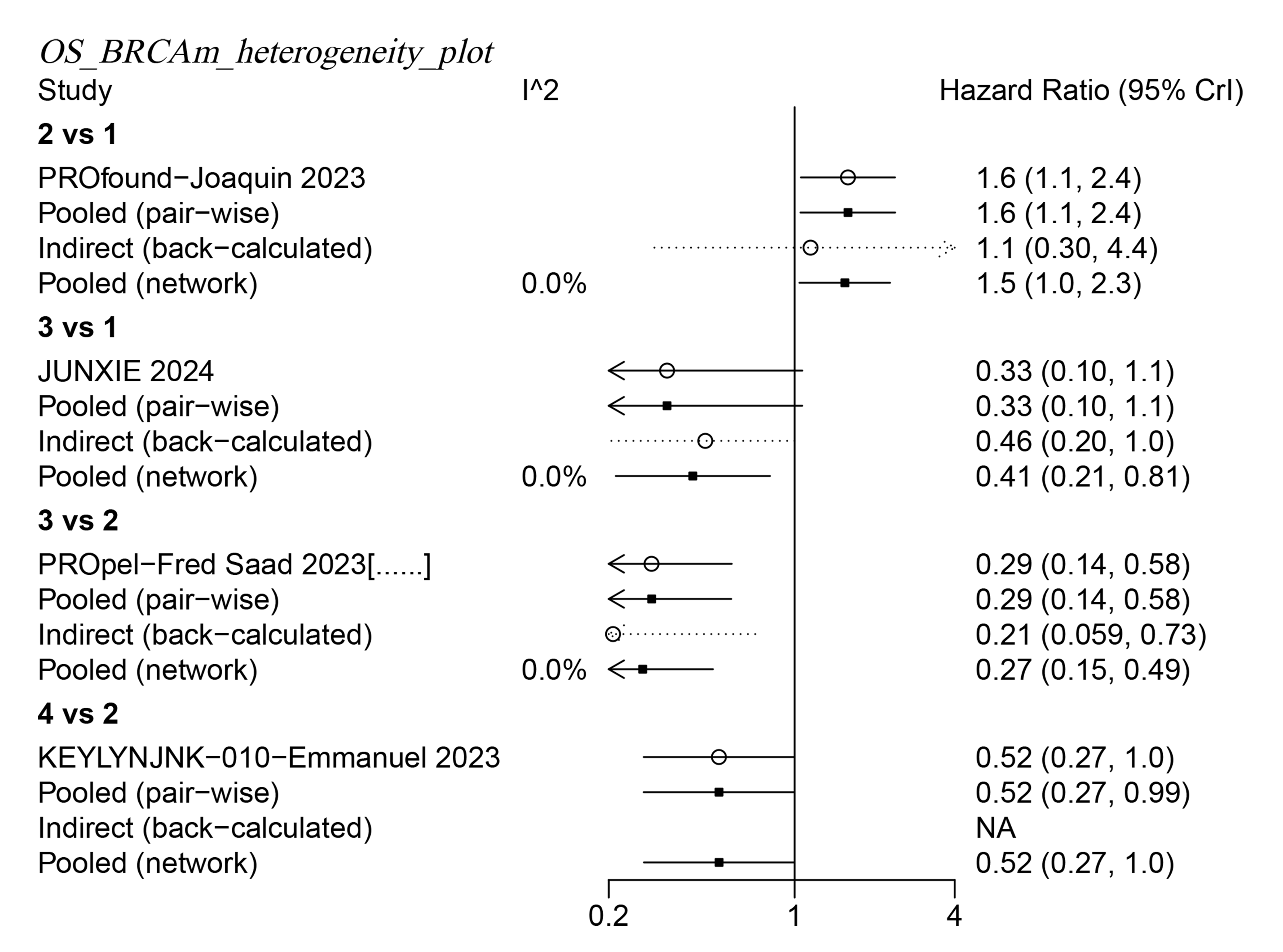


**Supplementary Figure 7** OS BRCAm heterogeneity plot.


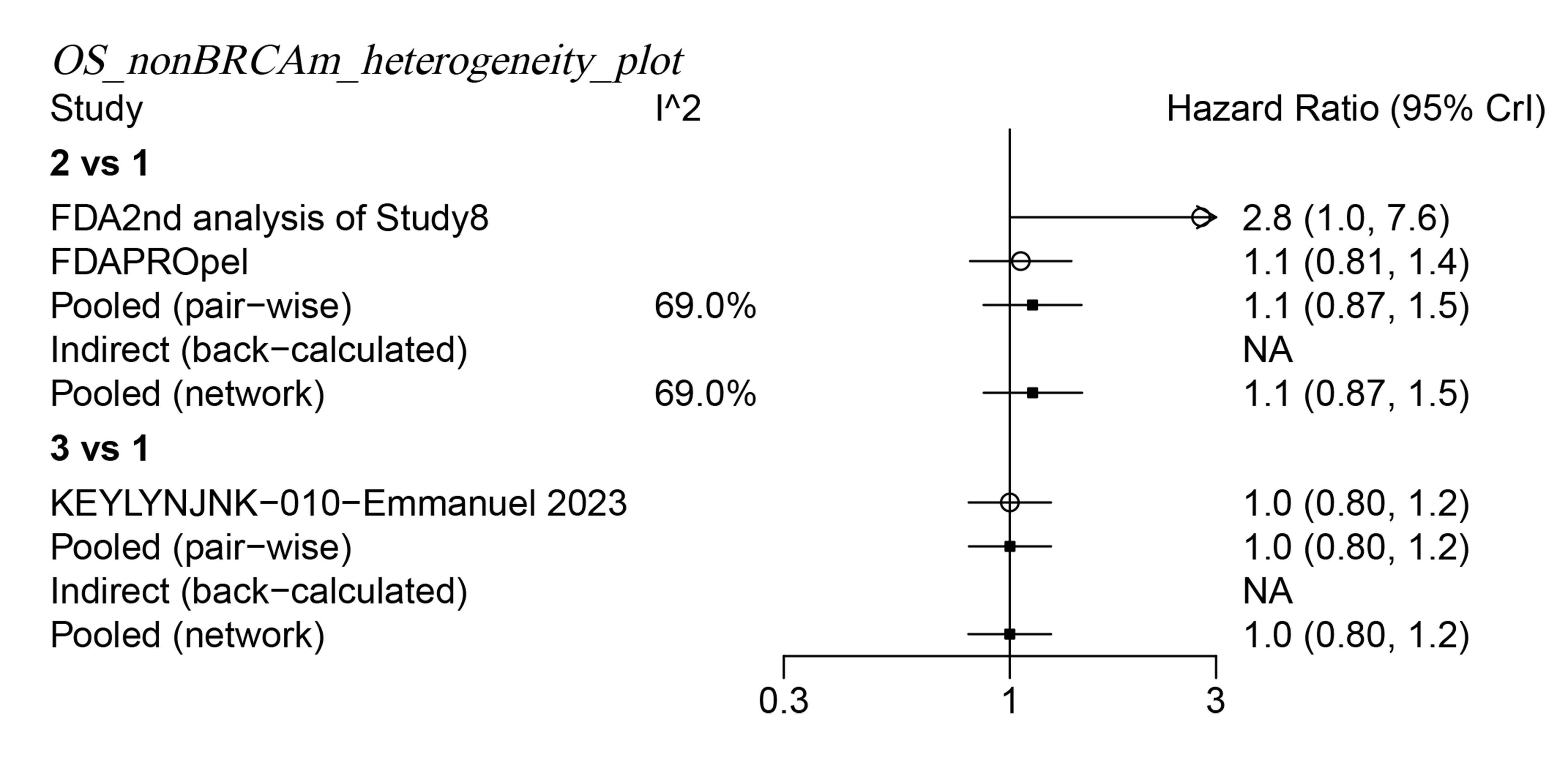


**Supplementary Figure 8** OS nonBRCAm heterogeneity plot.


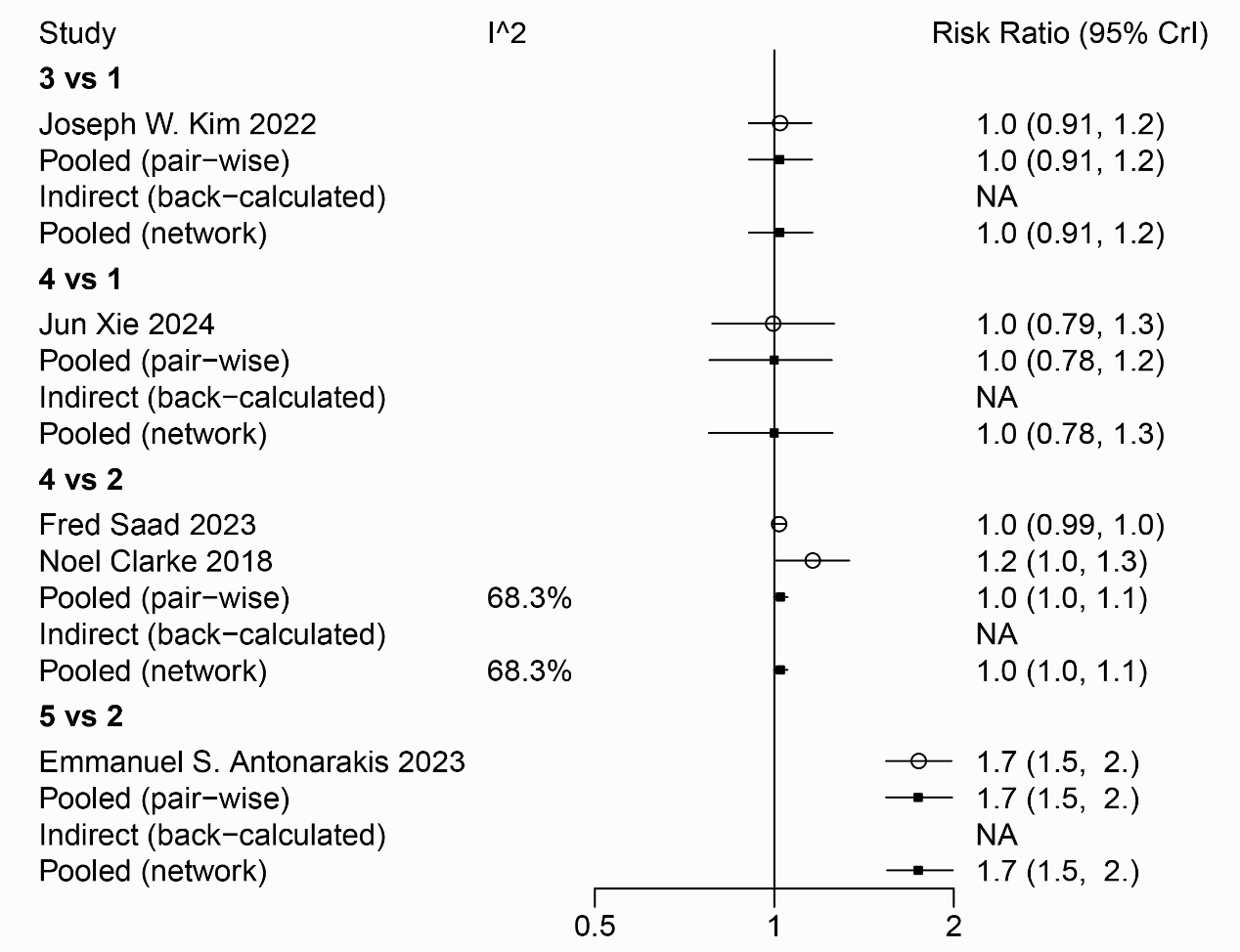


**Supplementary Figure 9** AE heterogeneity plot.


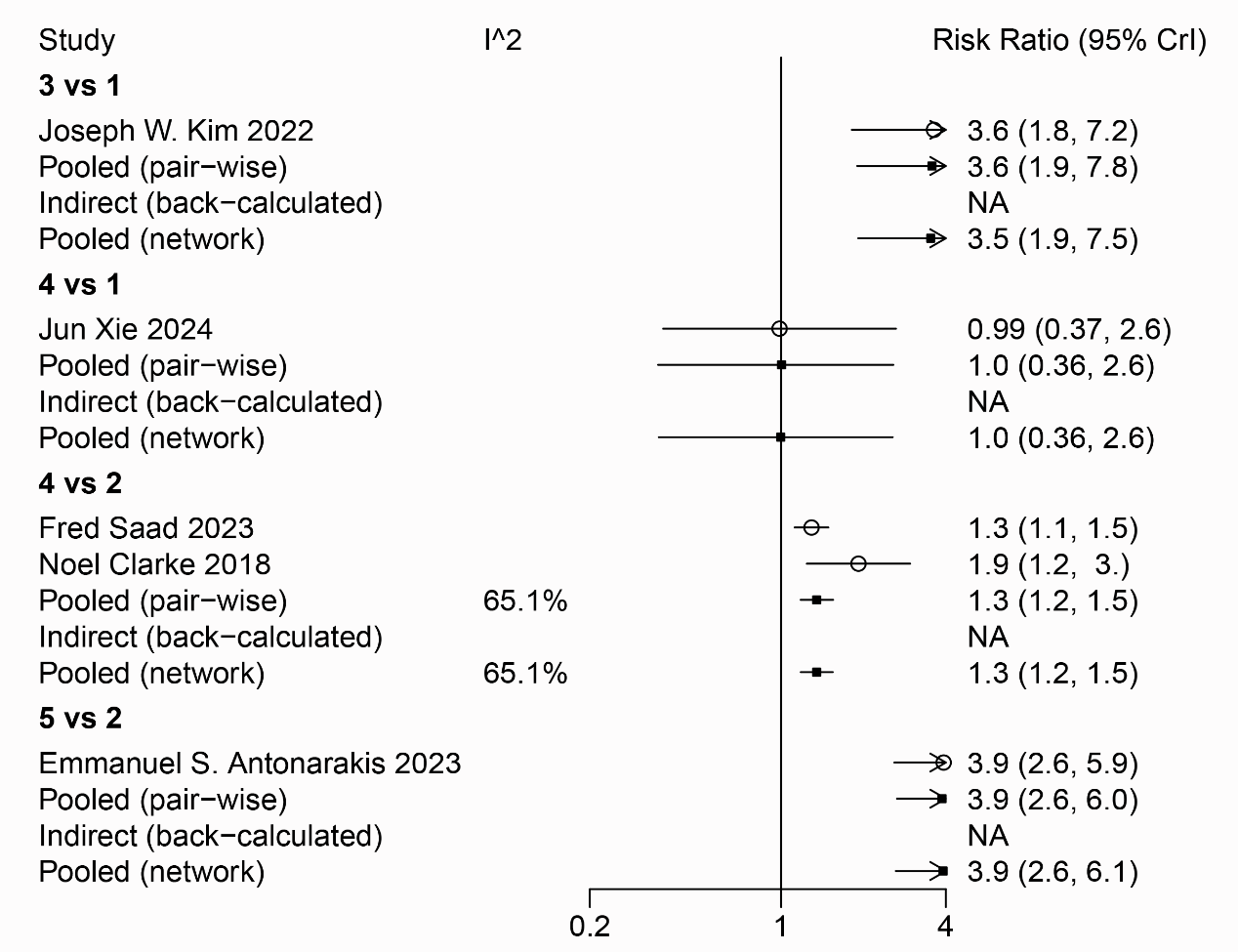


**Supplementary Figure 10** SAE heterogeneity plot.


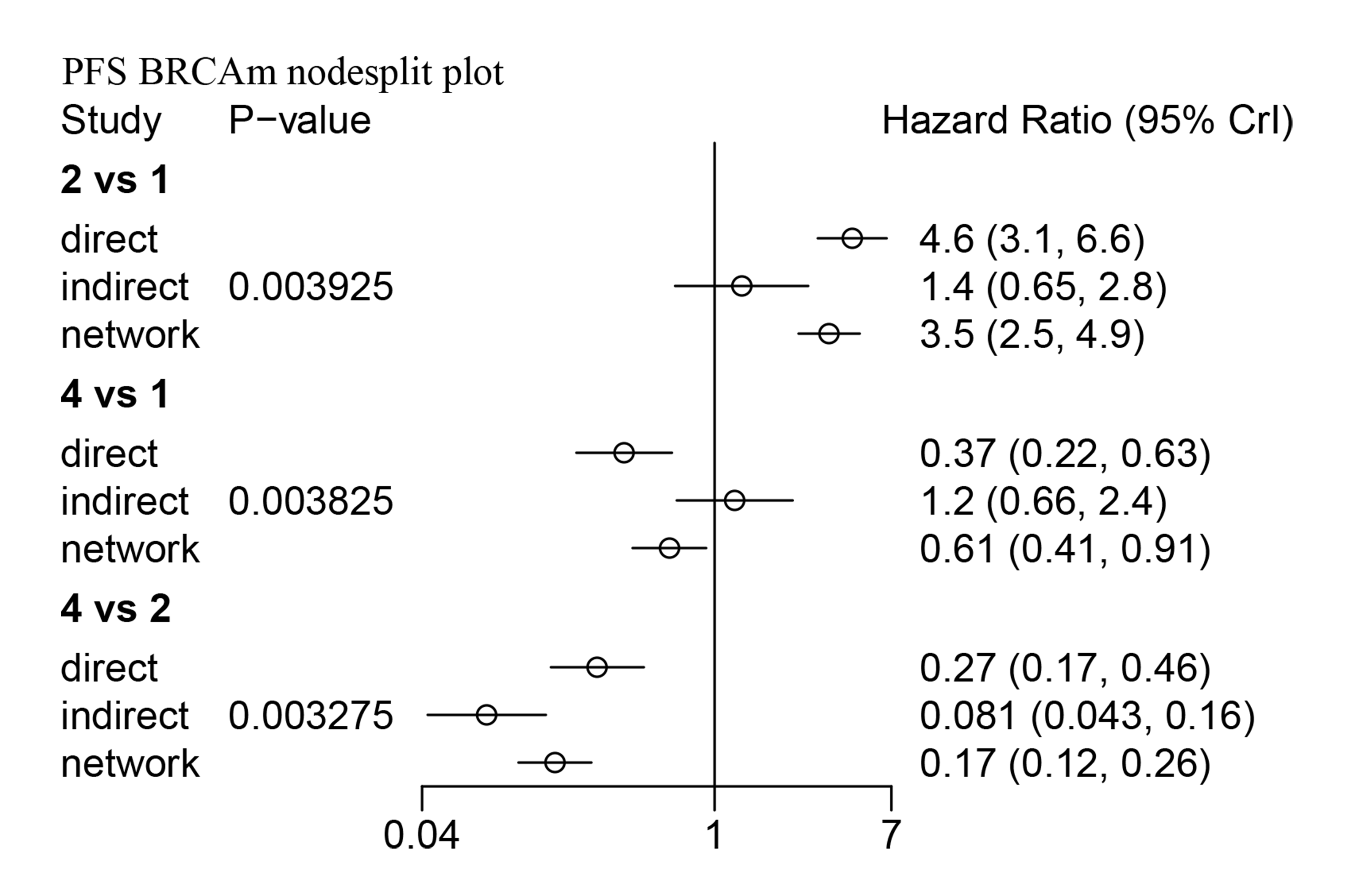


**Supplementary Figure 11** PFS BRCAm nodesplit plot.


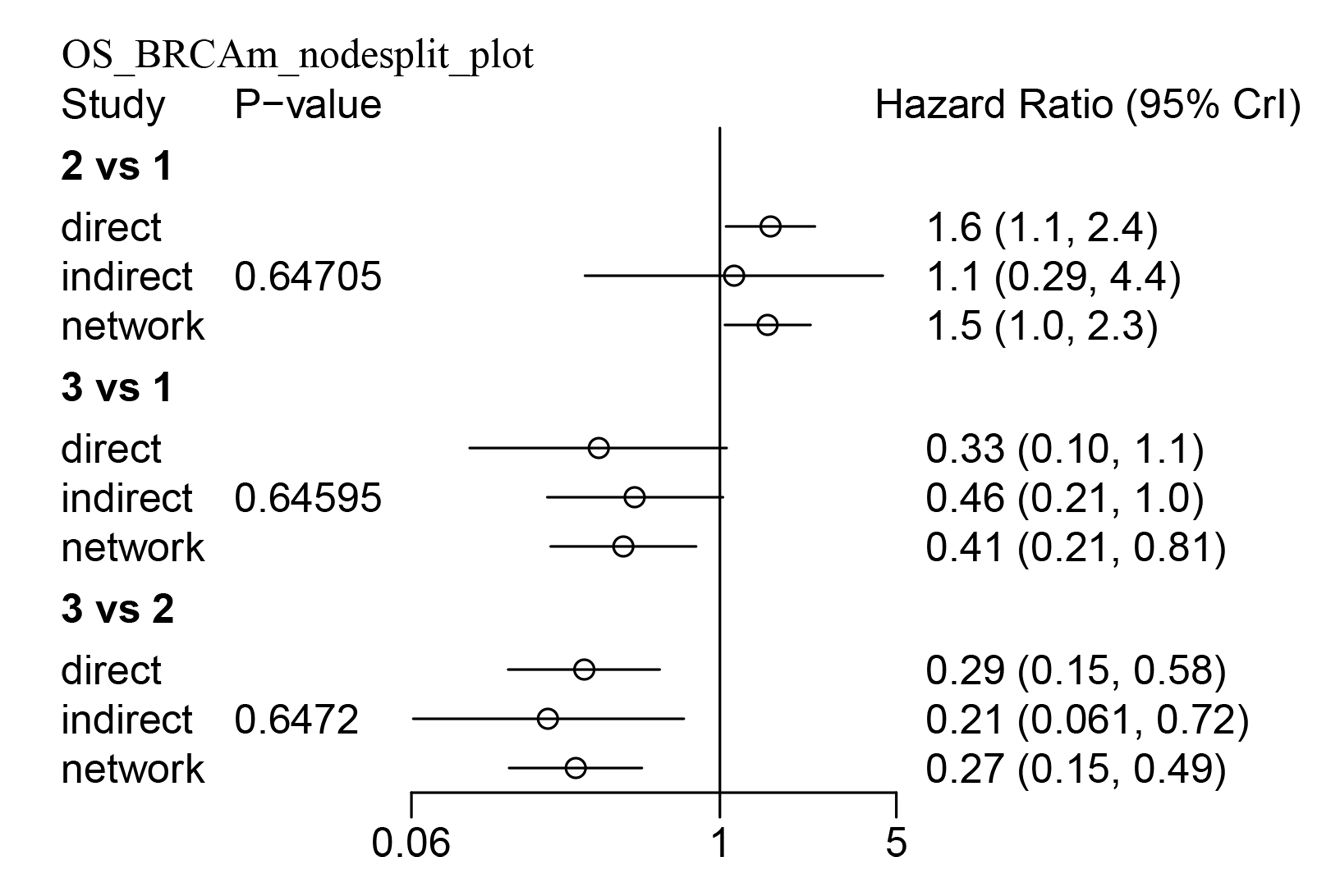


**Supplementary Figure 12** OS_BRCAm_nodesplit_plot.


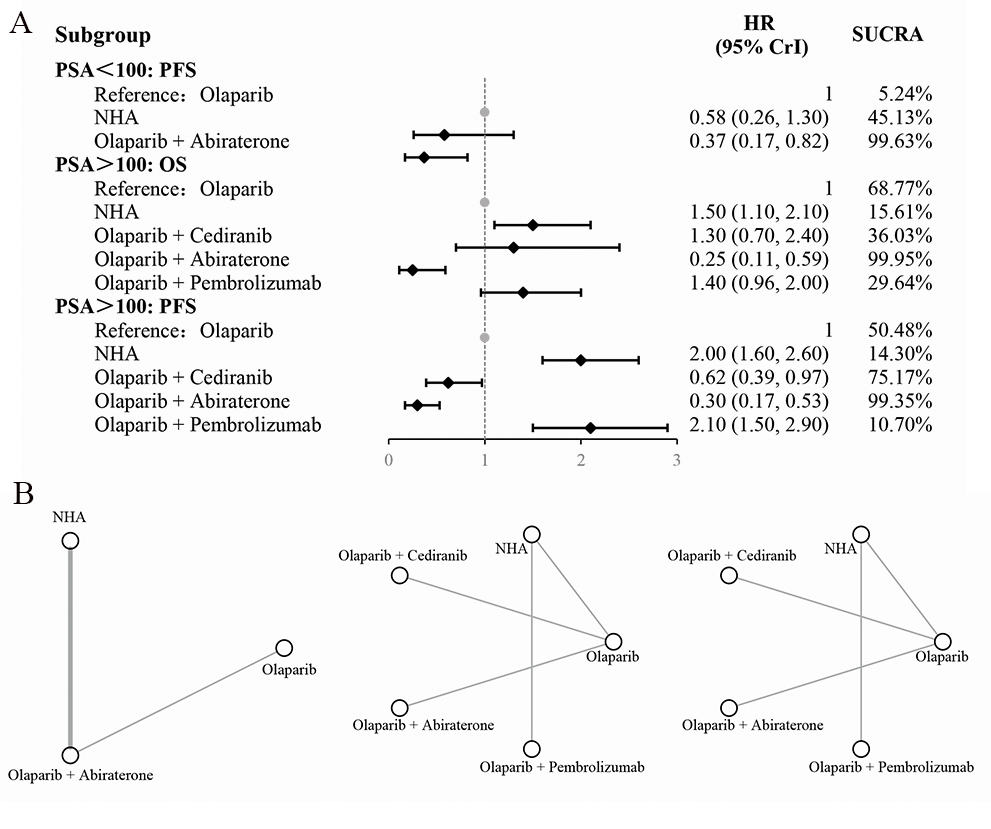


**Supplementary Figure 13** A) Forest plot and SUCRA PSA subgroups; B) Network plots in 1)PSA of PSA＜100,2)OS of PSA＞100,3)PFS of PSA＞100.


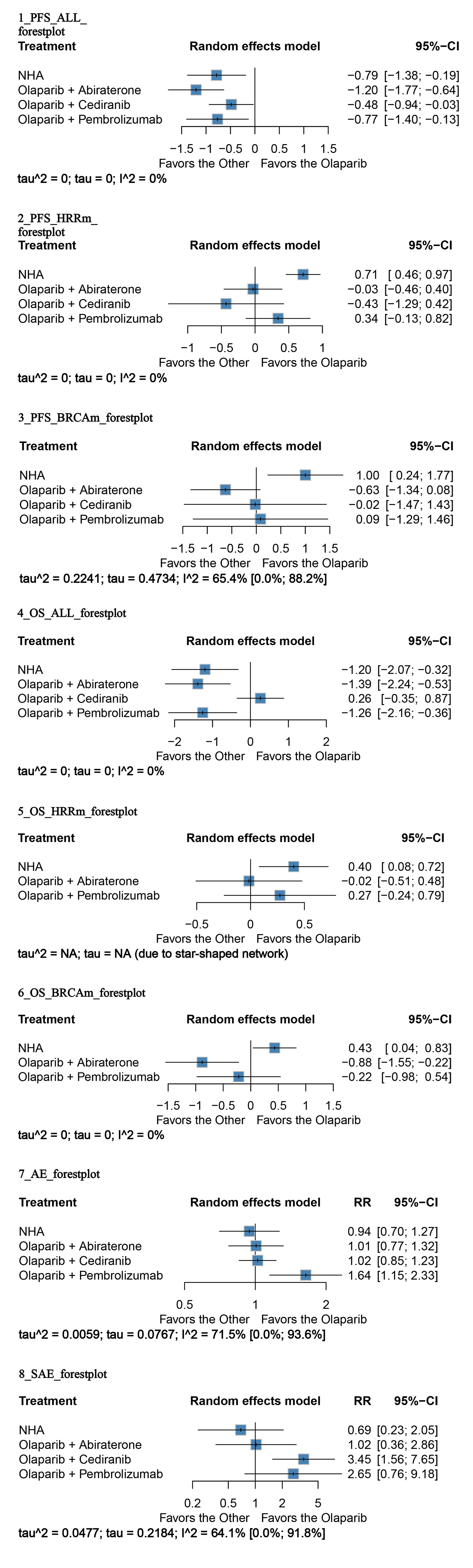


**Supplementary Figure 14** Frequentist Network Meta-Analysis Plot (with emphasis on Tau-squared)
